# Supplementary material for: Fairness and bias correction in machine learning for depression prediction across four study populations
Source: Sci Rep. 2024 Apr 3;14:7848. doi: 10.1038/s41598-024-58427-7 (PMC10991528; doi:10.1038/s41598-024-58427-7)
Supplement: Supplementary file 1 — Supplementary Information. [file 41598_2024_58427_MOESM1_ESM.pdf]

Supplementary Material

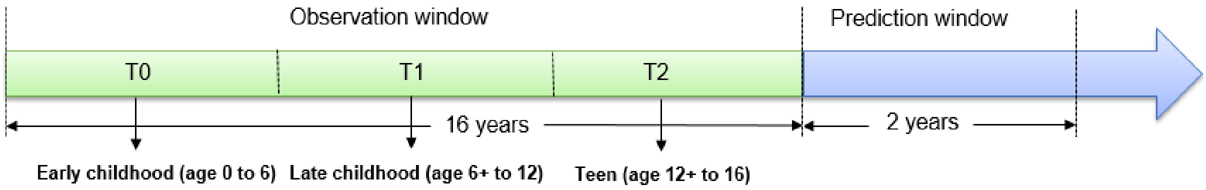

Supplementary Figure 1. Study design (LONGSCAN). The figure illustrates how we define the observation and prediction window. We aggregate each feature across each stage of child development (max) except for static features and time-varying demographic variables.

Supplementary Table 1. Protected attributes and other relevant features of the used datasets.

Subgroup	Attribute	LONGSCAN	FUUS	NHANES	UKB
	No. of subjects	911	4,184	36,259	461,033
	No. of features	47	62	86	143
Sex, % (Depression rate, %)	Male	44.2 (19.4)	42.6 (12.2)	49.1 (6.5)	46.6 (3.2)
	Female	55.8 (36.0)	57.4 (12.9)	50.9 (10.9)	53.4 (4.5)
Age (%); Depression rate (%)	0–20 years	-	-	7.5 (7.1)	-
	20–40 years	-	-	31.7 (8.2)	1.1 (4.2)
	40–60 years	-	-	31.1 (10.8)	59.9 (4.0)
	60–80 years	-	-	24.1 (8.0)	39.0 (3.8)
	>80 years	-	-	5.7 (5.7)	-
Ethnicity (%); Depression rate (%)	White	24.7 (35.1)	-	41.9 (8.6)	93.9 (3.9)
	Mexican	-	-	16.2 (8.6)	-
	Other Hispanic	-	-	9.5 (11.8)	-
	Black	55.9 (24.4)	-	21.9 (8.9)	1.7 (3.2)
	Asian	-	-	-	2.4 (3.1)
	Other/Multiracial	19.4 (32.8)	-	10.6 (6.2)	1.5 (4.7)
Nationality (%); Depression rate (%)	French	-	92.4 (12.3)	-	-
	Foreigner	-	7.6 (16.2)	-	-
Income (%); Depression rate (%)	Low-income	-	-	36.6 (13.3)	39.9 (4.9)
	High-income	-	-	63.4 (6.1)	44.7 (2.8)

Subgroup	Attribute	LONGSCAN	FUUS	NHANES	UKB
Qualifications (%); Depression rate (%)	Level 0	-	-	5.9 (7.0)	18.8 (5.4)
	Level 1	-	-	9.5 (12.9)	20.9 (3.9)
	Level 2	-	-	13.3 (13.2)	5.3 (5.0)
	Level 3	-	-	21.5 (9.3)	11.0 (3.6)
	Level 4	-	-	21.8 (8.9)	6.5 (4.4)
	Level 5	-	-	28.0 (3.9)	32.3 (2.9)
	Level 6	-	-	-	5.1 (4.0)
Diabetes (%); Depression rate (%)	Absence	-	-	-	94.4 (3.9)
	Presence	-	-	-	**5.1 (6.3)**
CVD (%); Depression rate (%)	Absence	-	-	-	76.8 (3.4)
	Presence	-	-	-	23.2 (5.6)

Qualification levels have different meanings in the NHANES and UKB datasets due to the difference in the United States and British school systems, more detailed in the Supplementary Table 10; The sum of all the percentages might not be equal to 100 as a result of missing data. Note that participants are all in the same age group in the LONGSCAN and FUUS datasets. The income attribute in the LONGSCAN is a time-varying variable. The FUUS dataset does not report ethnicity and income attributes.

Supplementary Table 2. Summary of all LONGSCAN input variables used in the study. All variables refer to the adolescents unless explicitly said. Time-varying variables can be observed at three different temporal points (T_0: age 0 to 6, T_1: age 6+ to 12, T_2: age 12+ to 16) considered in this study.

	Feature description	Type	Temporality
Demographic	1 Sex	Binary	Invariant
	2 Ethnicity	Categorical	Invariant
	3 Family income	Ordinal	T_0 , T_1, T_2
Lifestyles	4 Child’s smoking	Ordinal	T_1, T_2
	5 Child’s alcohol use	Ordinal	T_1, T_2
	6 Child’s substance use	Ordinal	T_1, T_2
Adverse Exposures	7 Emotional abuse	Binary	T_0 , T_1, T_2
	8 Physical abuse	Ordinal	T_0 , T_1, T_2
	9 Sexual abuse	Ordinal	T_0 , T_1, T_2
	10 Neglect	Ordinal	T_0 , T_1, T_2
	11 No. of emotional abuse	Numerical	Invariant
	12 No. of physical abuse	Numerical	Invariant

	13 No. of sexual abuse	Numerical	Invariant
	14 No. of neglect	Numerical	Invariant
	15 Child's serious illness or accident	Binary	T_0 , T_1, T_2
	16 Caregiver's alcohol use	Ordinal	T_0 , T_1, T_2
	17 Caregiver's substance use	Ordinal	T_1, T_2
	18 Caregiver's maltreatment	Ordinal	T_0 , T_1, T_2
	19 Criminal behavior in household	Binary	T_0 , T_1, T_2
	20 Death of family members	Binary	T_0 , T_1, T_2
	21 Anxiety	Binary	T_2
	22 Major fears, phobias, panic attacks	Binary	T_2
	23 ADD (Attention Deficit Disorder) / ADHD (Attention Deficit Hyperactivity Disorder)	Binary	T_2

Supplementary Table 3. Summary of all FUUS input variables used in the study.

Feature description	Type
1 Age	Categorical
2 Sex	Binary
3 French nationality	Binary
4 Field of study	Categorical
5 Year of university	Categorical
6 Learning disabilities	Binary
7 Difficulty memorizing lessons	Binary
8 Professional objective	Binary
9 Informed about opportunities	Binary
10 Satisfed with living conditions	Binary
11 Living with a partner/child	Binary
12 Parental home	Binary
13 Having only one parent	Binary
14 At least one parent unemployed	Binary
15 Siblings	Binary
16 Long commute	Binary
17 Mode of transportation	Categorical
18 Financial difficulties	Binary
19 Grant	Binary

20 Additional income	Binary
21 Public health insurance	Binary
22 Private health insurance	Binary
23 Universal health coverage	Binary
24 Irregular rhythm of meals	Binary
25 Unbalanced meals	Binary
26 Eating junk food	Binary
27 On a diet	Binary
28 Irregular rhythm or unbalanced meals	Binary
29 Physical activity (3 levels)	Ordinal
30 Physical activity (2 levels)	Binary
31 Weight (kg)	Numerical
32 Height (cm)	Numerical
33 Overweight and obesity	Binary
34 Systolic blood pressure (mmHg)	Numerical
35 Diastolic blood pressure (mmHg)	Numerical
36 Prehypertension or hypertension	Binary
37 Heart rate (bpm)	Numerical
38 Abnormal heart rate	Binary
39 Distant visual acuity of right eye (score/10)	Numerical
40 Distant visual acuity of left eye (score/10)	Numerical
41 Close visual acuity of right eye (score/10)	Numerical
42 Close visual acuity of left eye (score/10)	Numerical
43 Decreased in distant visual acuity	Binary
44 Decreased in close visual acuity	Binary
45 Urinalysis (glycosuria)	Binary
46 Urinalysis (proteinuria)	Binary
47 Urinalysis (hematuria)	Binary
48 Urinalysis (leukocyturia)	Binary
49 Urinalysis (positive nitrite test)	Binary
50 Abnormal urinalysis	Binary
51 Vaccination up to date	Binary
52 Control examination needed	Binary
53 Cigarette smoker (5 levels)	Ordinal

54 Cigarette smoker (3 levels)	Ordinal
55 Drinker (3 levels)	Ordinal
56 Drinker (2 levels)	Binary
57 Binge drinking	Binary
58 Marijuana use	Binary
59 Other recreational drugs	Binary
60 Body Mass Index (BMI)	Numerical
61 Mean Arterial Pressure (MAP)	Numerical
62 Pulse Pressure	Numerical

Supplementary Table 4. Summary of all NHANES input variables used in the study.

	Feature description	Type
Demographic Data	1 gender<br>2 age<br>3 ethnicity<br>4 citizenship<br>5 education_level<br>6 marital_status<br>7 household_size<br>8 pregnant<br>9 birth_place<br>10 veteran<br>11 household_income	1 Categorical<br>2 Numerical<br>3 Categorical<br>4 Categorical<br>5 Categorical<br>6 Categorical<br>7 Categorical<br>8 Categorical<br>9 Categorical<br>10 Categorical<br>11 Categorical
Medical Conditions	12 asthma<br>13 asthma_onset<br>14 asthma_currently<br>15 asthma_emergency<br>16 anaemia<br>17 ever_overweight<br>18 blood_transfusion<br>19 arthritis<br>20 heart_failure<br>21 heart_disease<br>22 angina<br>23 heart_attack<br>24 stroke<br>25 emphysema<br>26 bronchitis<br>27 liver_condition<br>28 thyroid_problem<br>29 bronchitis_currently<br>30 liver_condition_currently<br>31 thyroid_problem_currently<br>32 cancer<br>33 first_cancer_type<br>34 second_cancer_type<br>35 third_cancer_type<br>36 fourth_cancer_count<br>37 heart_attack_relative<br>38 asthma_relative<br>39 diabetes_relative<br>40 hay_fever<br>41 arthritis_onset<br>42 heart_failure_onset<br>43 heart_disease_onset<br>44 angina_onset<br>45 heart_attack_onset<br>46 stroke_onset<br>47 emphysema_onset<br>48 bronchitis_onset	12 Categorical<br>13 Numerical<br>14 Categorical<br>15 Categorical<br>16 Categorical<br>17 Categorical<br>18 Categorical<br>19 Categorical<br>20 Categorical<br>21 Categorical<br>22 Categorical<br>23 Categorical<br>24 Categorical<br>25 Categorical<br>26 Categorical<br>27 Categorical<br>28 Categorical<br>29 Categorical<br>30 Categorical<br>31 Categorical<br>32 Categorical<br>33 Categorical<br>34 Categorical<br>35 Categorical<br>36 Categorical<br>37 Categorical<br>38 Categorical<br>39 Categorical<br>40 Categorical<br>41 Numerical<br>42 Numerical<br>43 Numerical<br>44 Numerical<br>45 Numerical<br>46 Numerical<br>47 Numerical<br>48 Numerical

	49 liver_condition_onset<br>50 thyroid_problem_onset<br>51 cancer_onset<br>52 arthritis_type<br>53 first_cancer_count<br>54 second_cancer_count<br>55 third_cancer_count	49 Numerical<br>50 Numerical<br>51 Numerical<br>52 Categorical<br>53 Categorical<br>54 Categorical<br>55 Categorical
Body Measures	56 weight<br>57 height<br>58 BMI	56 Numerical<br>57 Numerical<br>58 Numerical
Occupation	59 full_time_work<br>60 work_type<br>61 time_in_current_job<br>62 out_of_work	59 Categorical<br>60 Categorical<br>61 Numerical<br>62 Categorical
Alcohol Use	63 drinks_per_occasion<br>64 lifetime_alcohol_consumption<br>65 drinks_past_year	63 Numerical<br>64 Numerical<br>65 Numerical
Drug Use	66 marijuana_use<br>67 marijuana_per_month<br>68 cocaine_use<br>69 cocaine_number_uses<br>70 cocaine_per_month<br>71 heroine_use<br>72 heronine_per_month<br>73 meth_use<br>74 meth_number_uses<br>75 meth_per_month<br>76 inject_drugs<br>77 rehab_program	66 Categorical<br>67 Numerical<br>68 Categorical<br>69 Numerical<br>70 Numerical<br>71 Categorical<br>72 Numerical<br>73 Categorical<br>74 Numerical<br>75 Numerical<br>76 Categorical<br>77 Categorical
Smoking Use	78 start_smoking_age<br>79 current_smoker<br>80 previous_cigarettes_per_day<br>81 current_cigarettes_per_day<br>82 days_quit_smoking<br>83 household_smokers	78 Numerical<br>79 Categorical<br>80 Numerical<br>81 Numerical<br>82 Numerical<br>83 Categorical
Prescription Medications	84 prescriptions_count<br>85 RXDDRUG (drug name)<br>86 RXDDAYS (number of days taken medicine)	84 Categorical<br>85 Categorical<br>86 Numerical

Supplementary Table 5. Summary of all UKB input variables used in the study.

	Feature description	Type
Demographic	1 Sex	Binary
	2 Ethnicity	Categorical
	3 Age at recruitment	Numerical
Education	4 Qualifications	Categorical
	5 Age completed full time education	Numerical
Employment	6 Current employment status	Categorical
Household	7 Average total household income before tax	Categorical
	8 Type of accommodation lived in	Categorical
	9 Own or rent accommodation lived in	Categorical

	10 Length of time at current address	Numerical
	11 Number in household	Numerical
	12 Number of vehicles in household	Categorical
Early life factors	13 Breastfed as a baby	Categorical
	14 Adopted as a child	Categorical
	15 Maternal smoking around birth	Categorical
Traumatic events	16 Felt hated by family member as a child	Categorical
	17 Physically abused by family as a child	Categorical
	18 Felt loved as a child	Categorical
	19 Sexually molested as a child	Categorical
	20 Someone to take to doctor when needed as a child	Categorical
	21 Avoided activities or situations because of previous stressful experience in past month	Categorical
	22 Repeated disturbing thoughts of stressful experience in past month	Categorical
	23 Felt very upset when reminded of stressful experience in past month	Categorical
	24 Belittlement by partner or ex-partner as an adult	Categorical
	25 Been in a confiding relationship as an adult	Categorical
	26 Physical violence by partner or ex-partner as an adult	Categorical
	27 Sexual interference by partner or ex-partner without consent as an adult	Categorical
	28 Able to pay rent/mortgage as an adult	Categorical
	29 Been in serious accident believed to be life-threatening	Categorical
	30 Been involved in combat or exposed to war-zone	Categorical
	31 Diagnosed with life-threatening illness	Categorical
	32 Victim of physically violent crime	Categorical
	33 Witnessed sudden violent death	Categorical
	34 Victim of sexual assault	Categorical
Alcohol use	35 Alcohol intake frequency	Categorical
	36 Alcohol drinker status	Categorical
	37 Ever had known person concerned about, or recommend reduction of, alcohol consumption	Categorical
	38 Ever been injured or injured someone else through drinking alcohol	Categorical
	39 Frequency of drinking alcohol	Categorical
Smoking	40 Current tobacco smoking	Categorical

	41 Past tobacco smoking	Categorical
	42 Smoking status	Categorical
Cannabis use	43 Ever taken cannabis	Categorical
Diet	44 Beef intake	Categorical
	45 Cheese intake	Categorical
	46 Cooked vegetable intake	Numerical
	47 Fresh fruit intake	Numerical
	48 Lamb/mutton intake	Categorical
	49 Pork intake	Categorical
	50 Poultry intake	Categorical
	51 Processed meat intake	Categorical
	52 Salad / raw vegetable intake	Numerical
	53 Salt added to food	Categorical
	54 Tea intake	Numerical
	55 Water intake	Numerical
	56 Dried fruit intake	Numerical
	57 Oily fish intake	Categorical
	58 Non-oily fish intake	Categorical
	59 Milk type used	Categorical
	60 Spread type	Categorical
	61 Bread intake	Numerical
	62 Bread type	Categorical
	63 Cereal intake	Numerical
	64 Cereal type	Categorical
	65 Coffee type	Categorical
	66 Major dietary changes in the last 5 years	Categorical
	67 Variation in diet	Categorical
	68 Non-butter spread type details	Categorical
	69 Never eat eggs, dairy, wheat, sugar	Categorical
Sleep	70 Daytime dozing / sleeping (narcolepsy)	Categorical
	71 Nap during day	Categorical
	72 Sleep duration	Numerical

	73 Sleeplessness / insomnia	Categorical
Medical history/conditions	74 Certain infectious and parasitic diseases	Binary
	75 Neoplasms	Binary
	76 Diseases of the blood and blood-forming organs and certain disorders involving the immune mechanism	Binary
	77 Endocrine, nutritional and metabolic diseases	Binary
	78 Other mental issue	Binary
	79 Diseases of the nervous system	Binary
	80 Diseases of the eye and adnexa	Binary
	81 Diseases of the ear and mastoid process	Binary
	82 Diseases of the circulatory system	Binary
	83 Diseases of the respiratory system	Binary
	84 Diseases of the digestive system	Binary
	85 Diseases of the skin and subcutaneous tissue	Binary
	86 Diseases of the musculoskeletal system and connective tissue	Binary
	87 Diseases of the genitourinary system	Binary
	88 Diabetes diagnosed by doctor	Categorical
	89 Number of self-reported non-cancer illnesses	Numerical
	90 Number of treatments/medications taken	Numerical
	91 Non-cancer illness year/age first occurred	Numerical
	92 Taking other prescription medications	Categorical
	93 Pregnant	Categorical
	94 Vascular/heart problems diagnosed by doctor	Categorical
	95 Blood clot, DVT, bronchitis, emphysema, asthma, rhinitis, eczema, allergy diagnosed by doctor	Categorical
	96 Medication for cholesterol, blood pressure, diabetes, or take exogenous hormones	Categorical
	97 Medication for cholesterol, blood pressure or diabetes	Categorical
	98 Ever sought or received professional help for mental distress	Categorical
	99 Ever suffered mental distress preventing usual activities	Categorical
	100 Circulatory system disorders (CVD)	Binary
Blood pressure	101 Diastolic blood pressure, automated reading	Numerical
	102 Systolic blood pressure, automated reading	Numerical
Body size measures	103 Body mass index (BMI)	Numerical

	104 Hip circumference	Numerical
	105 Waist circumference	Numerical
Body composition by impedance	106 Weight	Numerical
	107 Body fat percentage	Numerical
	108 Whole body fat mass	Numerical
	109 Whole body fat-free mass	Numerical
MET Scores	110 IPAQ activity group	Categorical
	111 Summed days activity	Numerical
	112 Summed minutes activity	Numerical
	113 Above moderate/vigorous recommendation	Categorical
	114 Above moderate/vigorous/walking recommendation	Categorical
	115 MET minutes per week for walking	Numerical
	116 MET minutes per week for moderate activity	Numerical
	117 MET minutes per week for vigorous activity	Numerical
Feelings/Tiredness	118 Ever depressed for a whole week	Categorical
	119 Ever unenthusiastic/disinterested for a whole week	Categorical
	120 Frequency of depressed mood in last 2 weeks	Categorical
	121 Frequency of tenseness / restlessness in last 2 weeks	Categorical
	122 Frequency of tiredness / lethargy in last 2 weeks	Categorical
	123 Frequency of unenthusiasm / disinterest in last 2 weeks	Categorical
	124 Illness, injury, bereavement, stress in last 2 years	Categorical
	125 Seen a psychiatrist for nerves, anxiety, tension or depression	Categorical
	126 Seen doctor (GP) for nerves, anxiety, tension or depression	Categorical
	127 Ever felt worried, tense, or anxious for most of a month or longer	Categorical
	128 Ever worried more than most people would in similar situation	Categorical
	129 Ever had prolonged loss of interest in normal activities	Categorical
	130 Recent easy annoyance or irritability	Categorical
	131 Recent feelings or nervousness or anxiety	Categorical
	132 Recent feelings of inadequacy	Categorical
	133 Recent trouble concentrating on things	Categorical
	134 Recent inability to stop or control worrying	Categorical
	135 Recent poor appetite or overeating	Categorical

	136 Recent feelings of foreboding	Categorical
	137 Recent lack of interest or pleasure in doing things	Categorical
	138 Recent trouble relaxing	Categorical
	139 Recent restlessness	Categorical
	140 Trouble falling or staying asleep, or sleeping too much	Categorical
	141 Recent changes in speed/amount of moving or speaking	Categorical
	142 Recent feelings of tiredness or low energy	Categorical
	143 Recent worrying too much about different things	Categorical

Supplementary Table 6. Implementation details of ML models and hyperparameter selection

Dataset	Model	Hyper-parameter
LONGSCAN	LR	l2-regularized, tune hyper-parameters of solver: ['newton-cg', 'lbfgs', 'liblinear'] and c_values: [100, 10, 1.0, 0.1, 0.01]
	XGB	{'max_depth': [1,3,5], 'learning_rate': [0.1,0.2,0.3], 'min_child_weight': [1,3,5], 'subsample': [0.3,0.5,0.7]}
FUUS	LR	l2-regularized, tune hyper-parameters of solver: ['newton-cg', 'lbfgs', 'liblinear'] and c_values: [100, 10, 1.0, 0.1, 0.01]
	XGB	{'max_depth': [1,3,5], 'learning_rate': [0.1,0.2,0.3], 'min_child_weight': [1,3,5], 'subsample': [0.3,0.5,0.7]}
NHANES	LR	l2-regularized, tune hyper-parameters of solver: ['newton-cg', 'lbfgs', 'liblinear'] and c_values: [100, 10, 1.0, 0.1, 0.01]
	XGB	{'max_depth': [1,3,5], 'learning_rate': [0.05, 0.15, 0.25], 'min_child_weight': [8,10,12], 'subsample': [0.3,0.5,0.7]}
UKB	LR	L2-regularized, solver='lbfgs', c_value = 1.0
	XGB	{'max_depth': [6], 'learning_rate' = [0.3], 'min_child_weight': [1], 'subsample': [1]}

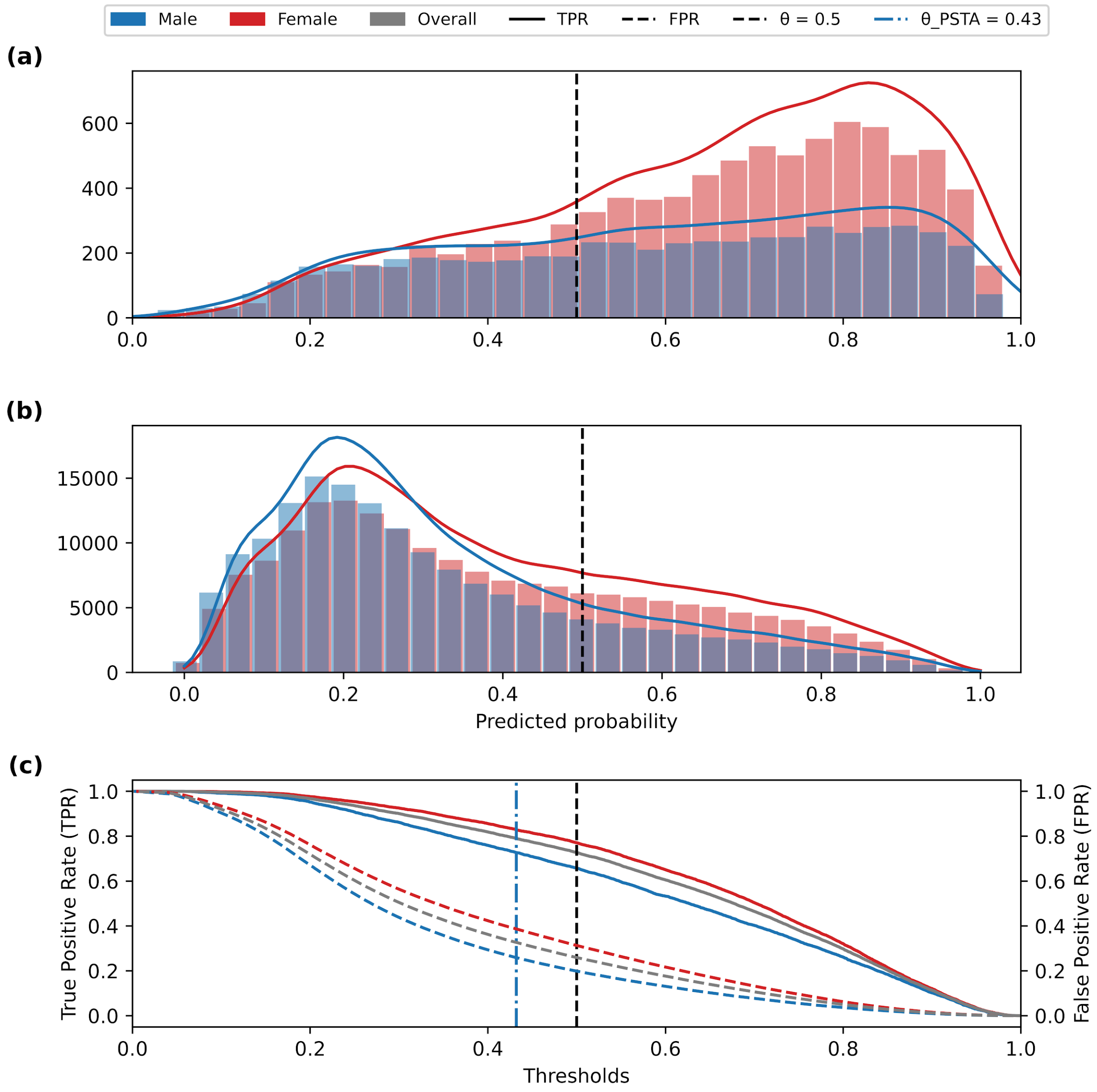

Supplementary Figure 2. **a,** Predicted Probability Distributions of LR Model on UKB Training Set by Sex for Positive Samples. **b,** Predicted Probability Distributions of LR Model on UKB Training Set by Sex for Negative Samples. **c,** TPR (solid lines) and FPR (dashed lines) for Male (blue, unprivileged) and Female (red) groups, and Overall (gray) for all possible thresholds. Vertical lines represent different thresholds: θ = 0.5 (black dashed line) and θ_PSTA = 0.43 for the unprivileged group (blue dash-dotted line).

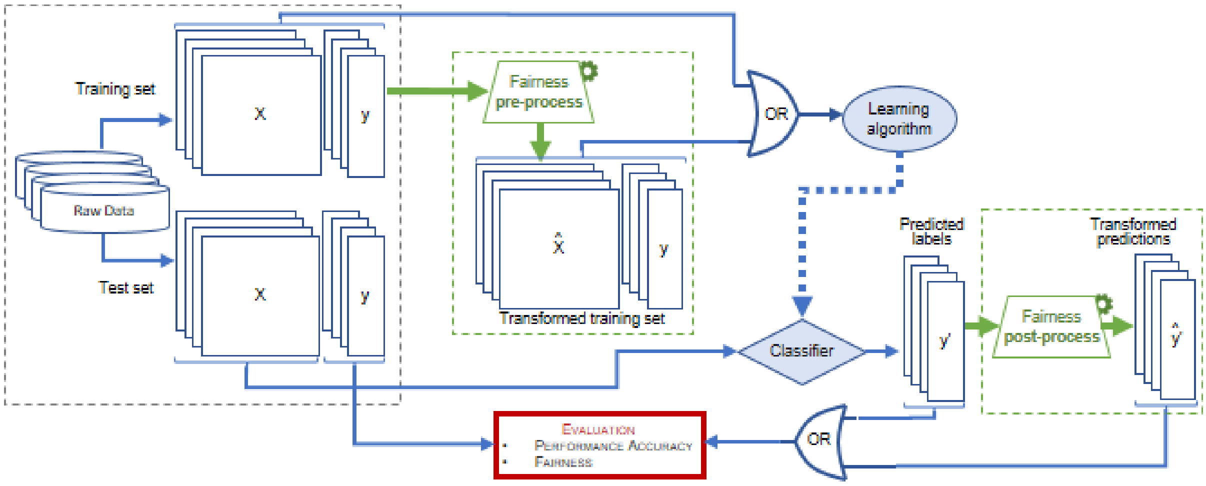

Supplementary Figure 3. The fairness ML pipeline used in this study.

Supplementary Table 7. Absolute and relative changes (in parentheses) in performance and fairness metrics before and after debiasing.

	Model	AUC-ROC	**BAcc**	**EOD**	AOD
LO*-Sex	LR-SUP	-0.041(-6.4%)	-0.058(-9.4%)	0.467(-113.7%)	0.434(-110.7%)
	LR-RW	-0.047(-7.3%)	-0.055(-8.9%)	0.452(-110.3%)	0.436(-111.3%)
	LR-DIR	-0.043(-6.7%)	-0.058(-9.3%)	0.433(-105.5%)	0.422(-107.8%)
	LR-CPP	-0.082(-12.7%)	-0.067(-10.9%)	0.417(-101.6%)	0.377(-96.3%)
	**LR-PSTA**	0.000(0.0%)	**-0.029(-4.7%)**	**0.303(-73.9%)**	0.302(-77.1%)
	XGB-SUP	-0.031(-4.9%)	-0.046(-7.4%)	0.439(-82.5%)	0.403(-88.2%)
	XGB-RW	-0.047(-7.3%)	-0.059(-9.5%)	0.477(-89.7%)	0.420(-91.8%)
	XGB-DIR	-0.042(-6.5%)	-0.061(-9.9%)	0.501(-94.2%)	0.459(-100.4%)
	XGB-CPP	-0.090(-13.9%)	-0.103(-16.6%)	0.596(-111.9%)	0.516(-112.9%)
	**XGB-PSTA**	0.000(0.0%)	**-0.019(-3.1%)**	**0.300(-56.4%)**	0.280(-61.3%)
FU*-Sex	LR-SUP	0.001(0.1%)	0.004(0.7%)	0.004(-6.1%)	0.007(-14.5%)
	**LR-RW**	0.001(0.2%)	**0.004(0.6%)**	**0.032(-45.7%)**	0.035(-77.4%)
	LR-DIR	-0.002(-0.3%)	0.003(0.4%)	0.013(-18.4%)	0.016(-35.5%)
	LR-CPP	-0.028(-4.2%)	-0.014(-2.2%)	0.039(-54.8%)	0.029(-63.6%)
	LR-PSTA	0.000(0.0%)	0.001(0.1%)	0.049(-69.1%)	0.047(-103.3%)
	XGB-SUP	0.005(0.8%)	0.006(0.9%)	0.015(-40.8%)	0.005(-409.7%)
	XGB-RW	0.001(0.2%)	-0.003(-0.4%)	-0.029(81.6%)	-0.011(884.6%)
	XGB-DIR	-0.015(-2.2%)	-0.008(-1.3%)	-0.010(27.9%)	-0.020(1538.1)
	XGB-CPP	-0.018(-2.7%)	-0.012(-2.0%)	0.013(-35.0%)	0.001(-90.8%)

	**XGB-PSTA**	0.000(0.0%)	**0.000(0.0%)**	**0.035(-97.9%)**	0.035(-2768.5)
NH*-Sex	LR-SUP	-0.001(-0.1%)	-0.001(-0.1%)	0.039(-23.1%)	0.035(-22.0%)
	**LR-RW**	-0.006(-0.8%)	**-0.006(-0.9%)**	**0.168(-100.4%)**	0.162(-100.4%)
	LR-DIR	-0.002(-0.3%)	-0.002(-0.3%)	0.094(-56.0%)	0.090(-56.0%)
	LR-CPP	-0.061(-7.8%)	-0.026(-3.7%)	0.129(-76.9%)	0.092(-57.3%)
	LR-PSTA	0.000(0.0%)	-0.003(-0.4%)	0.110(-65.7%)	0.102(-63.3%)
	XGB-SUP	0.001(0.1%)	-0.001(-0.2%)	0.037(-21.5%)	0.036(-22.0%)
	**XGB-RW**	-0.004(-0.5%)	**-0.006(-0.8%)**	**0.146(-83.4%)**	0.139(-85.5%)
	XGB-DIR	-0.002(-0.3%)	-0.003(-0.4%)	0.113(-64.8%)	0.111(-68.2%)
	XGB-CPP	-0.060(-7.5%)	-0.026(-3.6%)	0.123(-70.5%)	0.088(-54.0%)
	XGB-PSTA	0.000(0.0%)	-0.003(-0.4%)	0.092(-52.7%)	0.085(-52.5%)
UK*-Sex	LR-SUP	0.000(0.0%)	0.000(0.0%)	0.003(-2.6%)	0.003(-2.6%)
	**LR-RW**	-0.002(-0.3%)	**-0.003(-0.4%)**	**0.093(-83.0%)**	0.085(-74.7%)
	LR-DIR	0.000(0.0%)	0.000(0.0%)	0.015(-13.0%)	0.013(-11.4%)
	LR-CPP	-0.037(-4.6%)	-0.015(-2.1%)	0.076(-67.9%)	0.054(-47.2%)
	LR-PSTA	0.000(0.0%)	0.000(0.0%)	0.069(-61.2%)	0.062(-54.4%)
	XGB-SUP	0.004(0.5%)	0.002(0.2%)	0.004(-3.8%)	0.003(-2.9%)
	XGB-RW	0.003(0.3%)	0.001(0.1%)	0.063(-56.1%)	0.058(-50.4%)
	XGB-DIR	0.004(0.5%)	0.002(0.2%)	0.006(-5.6%)	0.005(-4.6%)
	XGB-CPP	-0.029(-3.7%)	-0.012(-1.6%)	0.071(-63.1%)	0.050(-44.0%)
	**XGB-PSTA**	0.004(0.5%)	**0.002(0.3%)**	**0.072(-64.1%)**	0.064(-55.6%)
LO*-Ethnicity	LR-SUP	-0.006(-0.9%)	-0.010(-1.6%)	0.154(-72.7%)	0.174(-92.4%)
	LR-RW	-0.007(-1.1%)	-0.018(-2.9%)	0.163(-77.2%)	0.189(-100.4%)
	**LR-DIR**	0.000(-0.1%)	**0.004(0.7%)**	**0.129(-60.7%)**	0.157(-83.2%)
	LR-CPP	-0.110(-17.1%)	-0.064(-10.3%)	0.343(-162.0%)	0.283(-150.5%)
	LR-PSTA	0.000(0.0%)	-0.015(-2.4%)	0.108(-50.8%)	0.120(-63.7%)
	XGB-SUP	0.000(0.0%)	-0.006(-0.9%)	0.007(-4.6%)	0.047(-31.9%)
	XGB-RW	-0.007(-1.1%)	-0.020(-3.3%)	0.121(-82.2%)	0.174(-118.5%)
	XGB-DIR	0.003(0.4%)	0.006(1.0%)	0.017(-11.5%)	0.076(-51.8%)
	XGB-CPP	-0.107(-16.4%)	-0.064(-10.3%)	0.376(-255.3%)	0.308(-210.6%)
	**XGB-PSTA**	0.000(0.0%)	**-0.011(-1.8%)**	**0.128(-86.8%)**	0.120(-82.0%)

FU*-Nationality	LR-SUP	0.001(0.2%)	0.004(0.6%)	-0.009(4.9%)	-0.001(0.6%)
	LR-RW	-0.001(-0.1%)	0.001(0.1%)	0.100(-51.4%)	0.126(-77.4%)
	LR-DIR	-0.004(-0.7%)	-0.023(-3.8%)	0.098(-50.5%)	0.084(-51.4%)
	LR-CPP	-0.015(-2.3%)	-0.007(-1.1%)	0.170(-87.6%)	0.128(-78.3%)
	**LR-PSTA**	0.000(0.0%)	**0.001(0.1%)**	**0.106(-54.4%)**	0.137(-84.3%)
	XGB-SUP	0.000(0.0%)	-0.001(-0.1%)	0.000(0.0%)	-0.001(0.9%)
	XGB-RW	0.003(0.5%)	-0.004(-0.7%)	0.022(-11.1%)	0.047(-29.3%)
	XGB-DIR	-0.022(-3.3%)	-0.032(-5.1%)	0.137(-67.9%)	0.107(-67.0%)
	XGB-CPP	-0.008(-1.2%)	-0.008(-1.2%)	0.172(-85.1%)	0.121(-75.9%)
	**XGB-PSTA**	0.000(0.0%)	**-0.002(-0.3%)**	**0.174(-86.2%)**	0.167(-104.4%)
NH*-Ethnicity	LR-SUP	0.000(0.0%)	0.003(0.4%)	0.070(-53.7%)	0.087(-46.0%)
	LR-RW	-0.002(-0.2%)	0.000(0.0%)	0.149(-114.7%)	0.169(-89.2%)
	LR-DIR	-0.002(-0.2%)	0.000(0.0%)	0.149(-114.7%)	0.169(-89.2%)
	LR-CPP	-0.281(-35.4%)	-0.177(-24.7%)	0.110(-84.0%)	0.085(-45.0%)
	**LR-PSTA**	0.000(0.0%)	**-0.001(-0.1%)**	**0.077(-59.4%)**	0.074(-39.1%)
	XGB-SUP	-0.001(-0.1%)	-0.004(-0.5%)	0.097(-59.4%)	0.089(-48.9%)
	**XGB-RW**	-0.002(-0.3%)	**-0.006(-0.8%)**	**0.151(-92.6%)**	0.146(-80.5%)
	XGB-DIR	-0.001(-0.2%)	-0.003(-0.4%)	0.093(-57.1%)	0.097(-53.2%)
	XGB-CPP	-0.277(-35.1%)	-0.172(-24.1%)	0.087(-53.5%)	0.068(-37.4%)
	XGB-PSTA	0.000(0.0%)	0.000(0.0%)	0.111(-67.8%)	0.097(-53.6%)
UK*-Ethnicity	LR-SUP	-0.001(-0.1%)	-0.001(-0.1%)	-0.025(9.1%)	-0.032(18.0%)
	LR-RW	0.000(0.0%)	0.000(0.0%)	-0.016(5.8%)	-0.008(4.6%)
	LR-DIR	-0.001(-0.1%)	-0.008(-1.1%)	-0.030(10.8%)	-0.040(22.8%)
	LR-CPP	-0.297(-37.2%)	-0.225(-30.8%)	0.155(-56.0%)	0.112(-63.5%)
	**LR-PSTA**	0.000(0.0%)	**0.000(0.0%)**	**0.226(-81.3%)**	0.257(-145.3%)
	XGB-SUP	0.000(0.0%)	0.000(0.0%)	0.001(-0.2%)	-0.002(1.2%)
	XGB-RW	0.000(0.0%)	-0.001(-0.1%)	0.004(-1.3%)	0.001(-0.7%)
	XGB-DIR	-0.001(-0.1%)	-0.001(-0.2%)	0.007(-2.5%)	0.003(-1.9%)
	XGB-CPP	-0.301(-37.5%)	-0.226(-30.9%)	0.164(-57.2%)	0.122(-66.5%)
	**XGB-PSTA**	0.000(0.0%)	**0.000(0.0%)**	**0.228(-79.7%)**	0.241(-131.9%)
NH*-Age	LR-SUP	0.000(0.0%)	-0.002(-0.2%)	0.010(-3.8%)	-0.019(15.9%)

	LR-RW	-0.002(-0.2%)	-0.002(-0.3%)	0.166(-62.1%)	0.115(-93.8%)
	LR-DIR	-0.003(-0.4%)	-0.007(-0.9%)	0.088(-33.0%)	0.053(-43.8%)
	LR-CPP	-0.236(-29.8%)	-0.137(-19.0%)	0.241(-90.2%)	0.135(-110.6%)
	**LR-PSTA**	0.000(0.0%)	**0.000(0.1%)**	**0.199(-74.5%)**	0.140(-114.9%)
	XGB-SUP	-0.003(-0.4%)	-0.004(-0.5%)	0.073(-20.7%)	0.039(-19.9%)
	XGB-RW	-0.002(-0.2%)	-0.006(-0.8%)	0.185(-52.2%)	0.146(-74.5%)
	XGB-DIR	-0.011(-1.4%)	-0.010(-1.4%)	0.105(-29.5%)	0.087(-44.2%)
	XGB-CPP	-0.237(-30.0%)	-0.139(-19.4%)	0.274(-77.4%)	0.162(-82.6%)
	**XGB-PSTA**	0.000(0.0%)	**-0.001(-0.2%)**	**0.232(-65.4%)**	0.179(-91.4%)
UK*-Age	LR-SUP	0.000(0.0%)	0.000(0.0%)	0.003(-5.9%)	0.010(-27.6%)
	LR-RW	0.000(0.0%)	0.000(0.0%)	0.007(-12.6%)	0.009(-23.2%)
	LR-DIR	-0.148(-18.5%)	-0.141(-19.3%)	-0.003(5.9%)	0.001(-3.0%)
	LR-CPP	-0.262(-32.7%)	-0.146(-20.0%)	0.042(-76.3%)	0.030(-81.6%)
	**LR-PSTA**	0.000(0.0%)	**0.000(0.0%)**	**0.029(-52.5%)**	0.028(-77.5%)
	XGB-SUP	-0.001(-0.1%)	0.000(0.0%)	0.004(-5.7%)	0.010(-22.2%)
	XGB-RW	0.000(0.0%)	0.000(0.0%)	-0.009(12.0%)	0.000(0.7%)
	XGB-DIR	-0.198(-24.6%)	-0.193(-26.4%)	0.111(-153.3%)	0.106(-235.3%)
	XGB-CPP	-0.265(-33.0%)	-0.147(-20.1%)	0.041(-56.3%)	0.029(-64.0%)
	**XGB-PSTA**	0.000(0.0%)	**0.000(0.0%)**	**0.031(-43.1%)**	0.031(-68.2%)
NH*-Income	LR-SUP	-0.003(-0.4%)	-0.004(-0.6%)	0.094(-37.7%)	0.087(-33.3%)
	**LR-RW**	-0.010(-1.2%)	**-0.005(-0.7%)**	**0.156(-62.6%)**	0.153(-58.5%)
	LR-DIR	-0.004(-0.4%)	-0.005(-0.7%)	0.099(-39.7%)	0.092(-35.1%)
	LR-CPP	-0.085(-10.7%)	-0.040(-5.5%)	0.213(-85.7%)	0.163(-62.4%)
	LR-PSTA	0.000(0.0%)	-0.006(-0.8%)	0.133(-53.5%)	0.120(-46.1%)
	XGB-SUP	-0.003(-0.4%)	-0.006(-0.8%)	0.100(-34.9%)	0.101(-35.0%)
	**XGB-RW**	-0.011(-1.4%)	**-0.007(-1.0%)**	**0.236(-81.9%)**	0.233(-81.0%)
	XGB-DIR	-0.002(-0.3%)	-0.006(-0.9%)	0.122(-42.3%)	0.117(-40.8%)
	XGB-CPP	-0.089(-11.3%)	-0.041(-5.8%)	0.222(-77.0%)	0.169(-58.9%)
	XGB-PSTA	0.000(0.0%)	-0.003(-0.4%)	0.148(-51.3%)	0.128(-44.6%)
UK*-Income	LR-SUP	0.000(0.0%)	0.000(-0.1%)	0.016(-11.1%)	0.015(-10.4%)
	**LR-RW**	-0.005(-0.6%)	**-0.004(-0.5%)**	**0.153(-106.2%)**	0.137(-97.1%)

	LR-DIR	-0.001(-0.1%)	0.000(0.0%)	0.027(-18.9%)	0.024(-17.1%)
	LR-CPP	-0.143(-17.9%)	-0.062(-8.5%)	0.113(-78.3%)	0.082(-57.7%)
	LR-PSTA	0.000(0.0%)	0.000(-0.1%)	0.087(-60.5%)	0.075(-53.2%)
	XGB-SUP	0.000(0.0%)	0.000(0.0%)	0.015(-13.8%)	0.014(-11.8%)
	**XGB-RW**	-0.003(-0.4%)	**-0.001(-0.1%)**	**0.101(-90.3%)**	0.091(-77.0%)
	XGB-DIR	-0.001(-0.1%)	0.000(0.1%)	0.030(-27.1%)	0.027(-22.7%)
	XGB-CPP	-0.140(-17.4%)	-0.059(-8.1%)	0.094(-84.2%)	0.069(-58.5%)
	XGB-PSTA	0.000(0.0%)	0.000(0.0%)	0.067(-59.4%)	0.057(-48.0%)
NH*-Qualifications	LR-SUP	-0.001(-0.2%)	-0.001(-0.2%)	0.074(-22.4%)	0.056(-20.1%)
	LR-RW	-0.008(-1.0%)	-0.012(-1.7%)	0.226(-68.4%)	0.192(-69.6%)
	LR-DIR	-0.002(-0.2%)	-0.017(-2.3%)	0.101(-30.7%)	0.059(-21.3%)
	LR-CPP	-0.213(-26.9%)	-0.106(-14.8%)	0.246(-74.7%)	0.179(-64.9%)
	**LR-PSTA**	0.000(0.0%)	**-0.009(-1.2%)**	**0.251(-76.2%)**	0.219(-79.5%)
	XGB-SUP	-0.002(-0.2%)	-0.002(-0.3%)	0.047(-13.3%)	0.042(-14.7%)
	XGB-RW	-0.006(-0.7%)	-0.008(-1.1%)	0.245(-69.8%)	0.208(-72.5%)
	XGB-DIR	-0.003(-0.4%)	-0.011(-1.6%)	0.107(-30.4%)	0.073(-25.3%)
	XGB-CPP	-0.211(-26.7%)	-0.105(-14.6%)	0.249(-70.9%)	0.181(-63.1%)
	**XGB-PSTA**	0.000(0.0%)	**-0.007(-0.9%)**	**0.275(-78.3%)**	0.229(-79.9%)
UK*-Qualifications	LR-SUP	0.000(0.0%)	0.000(0.0%)	0.024(-19.4%)	0.023(-18.4%)
	LR-RW	0.000(0.0%)	0.000(0.0%)	0.001(-0.6%)	0.000(-0.4%)
	LR-DIR	0.000(0.0%)	-0.005(-0.7%)	0.015(-12.2%)	0.005(-4.1%)
	LR-CPP	-0.302(-37.7%)	-0.169(-23.1%)	0.089(-72.9%)	0.065(-52.8%)
	**LR-PSTA**	0.000(0.0%)	**0.000(0.0%)**	**0.070(-56.7%)**	0.061(-49.1%)
	XGB-SUP	0.000(0.0%)	-0.001(-0.1%)	0.005(-6.1%)	0.006(-6.8%)
	XGB-RW	0.000(0.0%)	0.000(0.0%)	0.044(-59.5%)	0.073(-88.2%)
	XGB-DIR	-0.001(-0.1%)	0.000(0.0%)	0.006(-7.7%)	0.004(-5.3%)
	XGB-CPP	-0.304(-37.9%)	-0.169(-23.1%)	0.064(-86.5%)	0.048(-57.6%)
	**XGB-PSTA**	0.000(0.0%)	**0.000(0.0%)**	**0.049(-66.7%)**	0.042(-50.7%)
UK*-Diabetes	LR-SUP	0.000(0.0%)	0.000(0.0%)	-0.003(3.2%)	-0.005(3.5%)
	LR-RW	-0.001(-0.2%)	-0.001(-0.2%)	0.122(-116.9%)	0.134(-103.1%)
	LR-DIR	-0.047(-5.9%)	-0.088(-12.0%)	0.006(-5.8%)	-0.002(1.9%)

	**LR-CPP**	-0.012(-1.5%)	**-0.005(-0.7%)**	**0.111(-106.5%)**	0.083(-64.1%)
	LR-PSTA	0.000(0.0%)	0.000(0.0%)	0.007(-6.9%)	0.007(-5.2%)
	XGB-SUP	0.000(0.0%)	0.000(0.0%)	0.000(0.0%)	0.000(0.0%)
	**XGB-RW**	-0.001(-0.1%)	**-0.001(-0.1%)**	**0.064(-74.7%)**	0.077(-66.4%)
	XGB-DIR	0.000(0.0%)	-0.001(-0.1%)	-0.001(1.5%)	0.000(-0.1%)
	XGB-CPP	-0.012(-1.5%)	-0.004(-0.6%)	0.096(-112.5%)	0.073(-63.6%)
	XGB-PSTA	0.000(0.0%)	0.000(0.0%)	0.006(-7.2%)	0.006(-5.1%)
UK*-CVD	LR-SUP	0.000(0.0%)	0.000(0.0%)	0.000(0.1%)	0.000(0.0%)
	**LR-RW**	-0.004(-0.5%)	**-0.004(-0.5%)**	**0.130(-109.0%)**	0.127(-96.9%)
	LR-DIR	0.000(0.0%)	0.000(0.0%)	0.015(-12.2%)	0.015(-11.4%)
	LR-CPP	-0.029(-3.7%)	-0.012(-1.7%)	0.110(-92.6%)	0.080(-61.0%)
	LR-PSTA	0.000(0.0%)	0.001(0.1%)	0.038(-32.2%)	0.035(-26.3%)
	XGB-SUP	0.000(0.0%)	0.000(0.1%)	0.004(-3.8%)	0.005(-4.6%)
	**XGB-RW**	-0.003(-0.4%)	**-0.001(-0.2%)**	**0.083(-88.5%)**	0.083(-76.4%)
	XGB-DIR	0.000(-0.1%)	0.000(0.0%)	0.015(-15.9%)	0.014(-12.5%)
	XGB-CPP	-0.026(-3.3%)	-0.011(-1.5%)	0.094(-100.4%)	0.068(-62.4%)
	XGB-PSTA	0.000(0.0%)	0.001(0.1%)	0.032(-33.9%)	0.028(-25.9%)

*LO, FU, NH, and UK represent the LONGSCAN, FUUS, NHANES, and UKB datasets, respectively.

Supplementary Table 8. AUC-ROC performance of LR and XGB classifiers after applying bias mitigation algorithms, reporting the best results. Note that this value remains unchanged after applying the PSTA technique.

Protected attribute	Dataset	Model	AUC-ROC*
Sex	LONGSCAN	LR - PSTA	0.644 [0.590-0.699]
		XGB - PSTA	0.649 [0.595-0.702]
	FUUS	LR - RW	0.672 [0.648-0.696]
		XGB - PSTA	0.660 [0.638-0.681]
	NHANES	LR - RW	0.786 [0.779-0.793]
		XGB - RW	0.785 [0.779-0.792]
	UKB	LR - RW	0.797 [0.792-0.803]
		XGB - PSTA	0.804 [0.798-0.809]
Ethnicity	LONGSCAN	LR - DIR	0.644 [0.588-0.699]
		XGB - PSTA	0.649 [0.595-0.702]
	NHANES	LR - PSTA	0.793 [0.785-0.800]
		XGB - RW	0.787 [0.779-0.795]
	UKB	LR - PSTA	0.800 [0.794-0.806]
		XGB - PSTA	0.804 [0.798-0.809]
Nationality	FUUS	LR - PSTA	0.671 [0.647-0.695]
		XGB - PSTA	0.660 [0.638-0.681]
Age	NHANES	LR - PSTA	0.793 [0.785-0.800]
		XGB - PSTA	0.789 [0.783-0.796]
	UKB	LR - PSTA	0.800 [0.794-0.806]
		XGB - PSTA	0.804 [0.798-0.809]
Income	NHANES	LR - RW	0.783 [0.774-0.792]
		XGB - RW	0.779 [0.769-0.788]
	UKB	LR - RW	0.795 [0.789-0.801]
		XGB - RW	0.800 [0.795-0.806]
Qualifications	NHANES	LR - DIR	0.791 [0.783-0.798]
		XGB - PSTA	0.789 [0.783-0.796]
	UKB	LR - PSTA	0.800 [0.794-0.806]
		XGB - PSTA	0.804 [0.798-0.809]
Diabetes	UKB	LR - CPP	0.788 [0.782-0.793]

		XGB - RW	0.803 [0.797-0.808]
CVD	UKB	LR - RW	0.796 [0.789-0.802]
		XGB - RW	0.801 [0.795-0.806]

*Mean [95%-confidence interval]

Supplementary Table 9. Predictive performance of LR classifiers learned without bias mitigation from the different datasets.

Dataset	BAcc*	AUC-ROC*
LONGSCAN	0.621 [0.577-0.664]	0.644 [0.590-0.699]
FUUS	0.615 [0.598-0.632]	0.671 [0.647-0.695]
NHANES	0.719 [0.711-0.727]	0.793 [0.785-0.800]
UKB	0.729 [0.725-0.734]	0.800 [0.794-0.806]

*Mean [95%-confidence interval]

Supplementary Table 10. Predictive performance of XGB classifiers learned without bias mitigation from the different datasets.

Dataset	BAcc*	AUC-ROC*
LONGSCAN	0.621 [0.578-0.664]	0.649 [0.595-0.702]
FUUS	0.618 [0.601-0.636]	0.660 [0.638-0.681]
NHANES	0.715 [0.708-0.721]	0.789 [0.783-0.796]
UKB	0.731 [0.724-0.737]	0.804 [0.798-0.809]

*Mean [95%-confidence interval]

Supplementary Table 11. Qualification levels.

Qualification levels	NHANES	UKB
Level 0	Refused/ Don’t know/ Missing	None of the below/Prefer not to answer/Missing
Level 1	Less than 9th grade	O levels/GCSEs or equivalent
Level 2	9-11th grade (Includes 12th grade with no diploma)	CSEs or equivalent
Level 3	High school Grad/GED or Equivalent	A levels/AS levels or equivalent
Level 4	Some college or AA degree	NVQ or HND or HNC or equivalent
Level 5	College Graduate or above	College or University degree
Level 6	–	Other professional qualifications eg: nursing, teaching

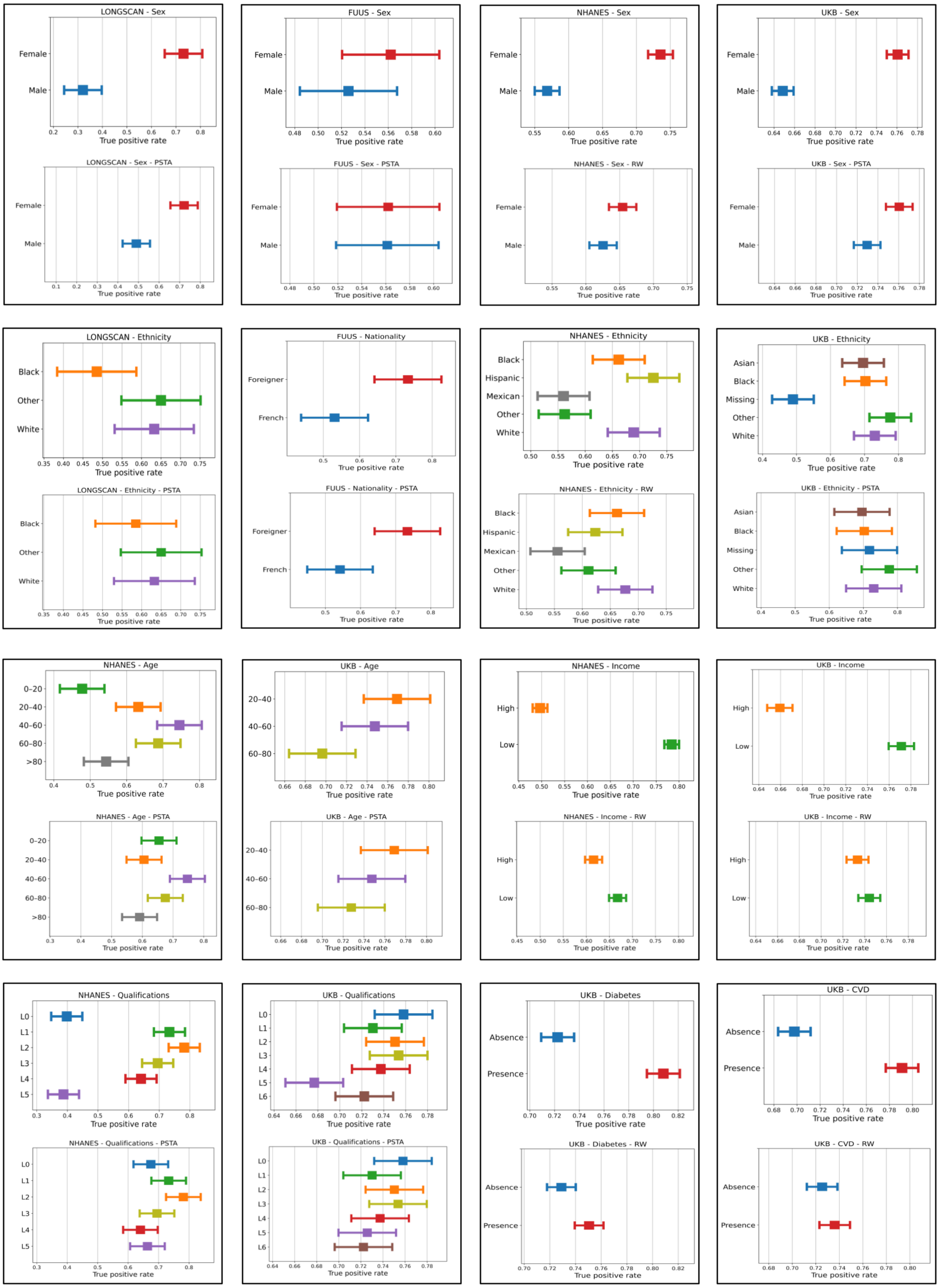

Supplementary Figure 4. Comparative analysis of group-specific TPRs for XGB classifiers: baseline and bias mitigation outcomes. Points represent mean TRP and error bars indicate 95% confidence intervals over k-fold cross validation. Each plot represents a Dataset-Protected attribute pair, with paired rows displaying base classifiers and debiased classifiers, reporting the best results among the tested bias mitigation algorithms.

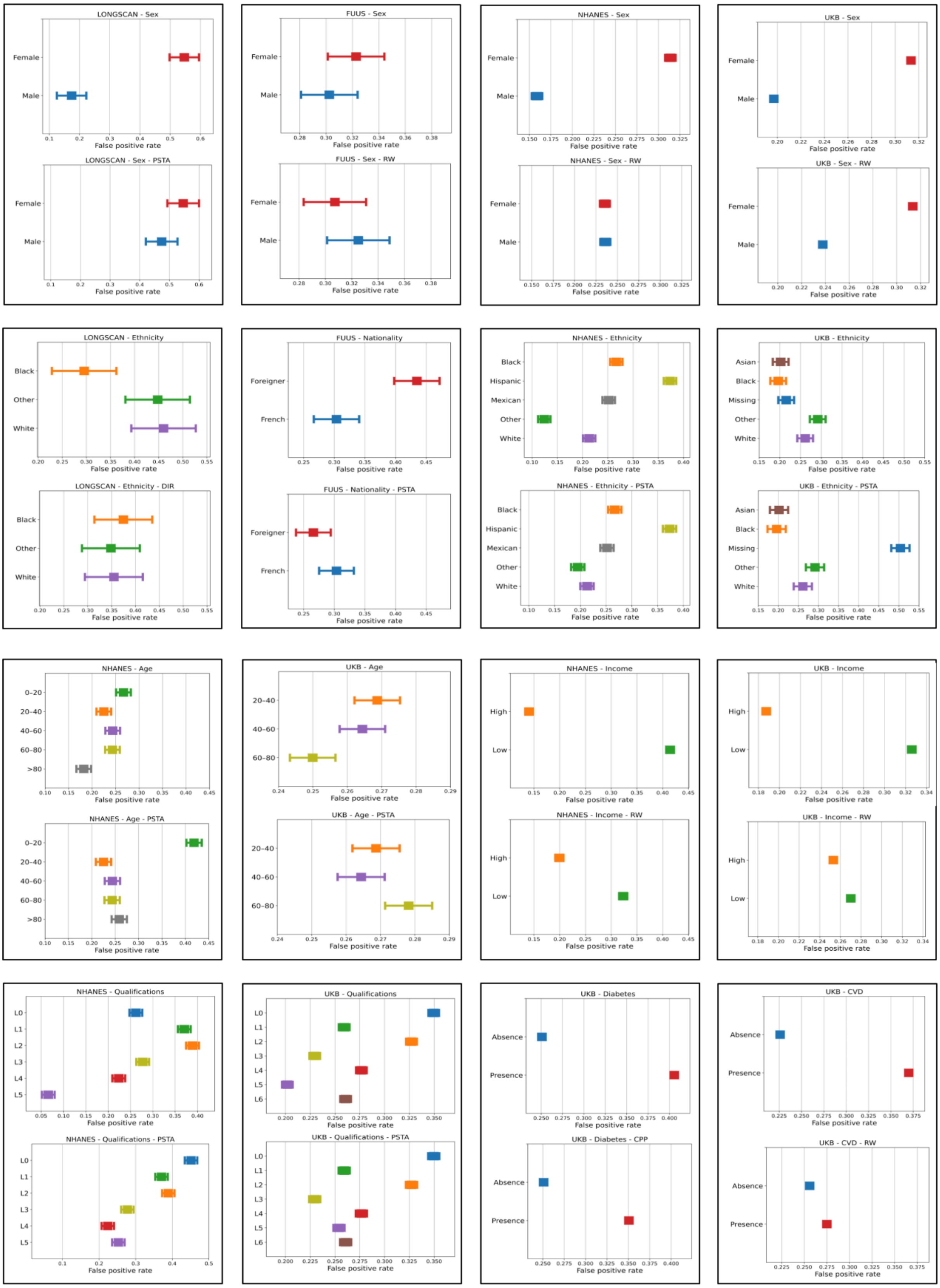

Supplementary Figure 5. Comparative analysis of group-specific FPRs for LR classifiers: baseline and bias mitigation outcomes. Points represent mean FRP and error bars indicate 95% confidence intervals over k-fold cross validation. Each plot represents a Dataset-Protected attribute pair, with paired rows displaying base classifiers and debiased classifiers, reporting the best results among the tested bias mitigation algorithms.

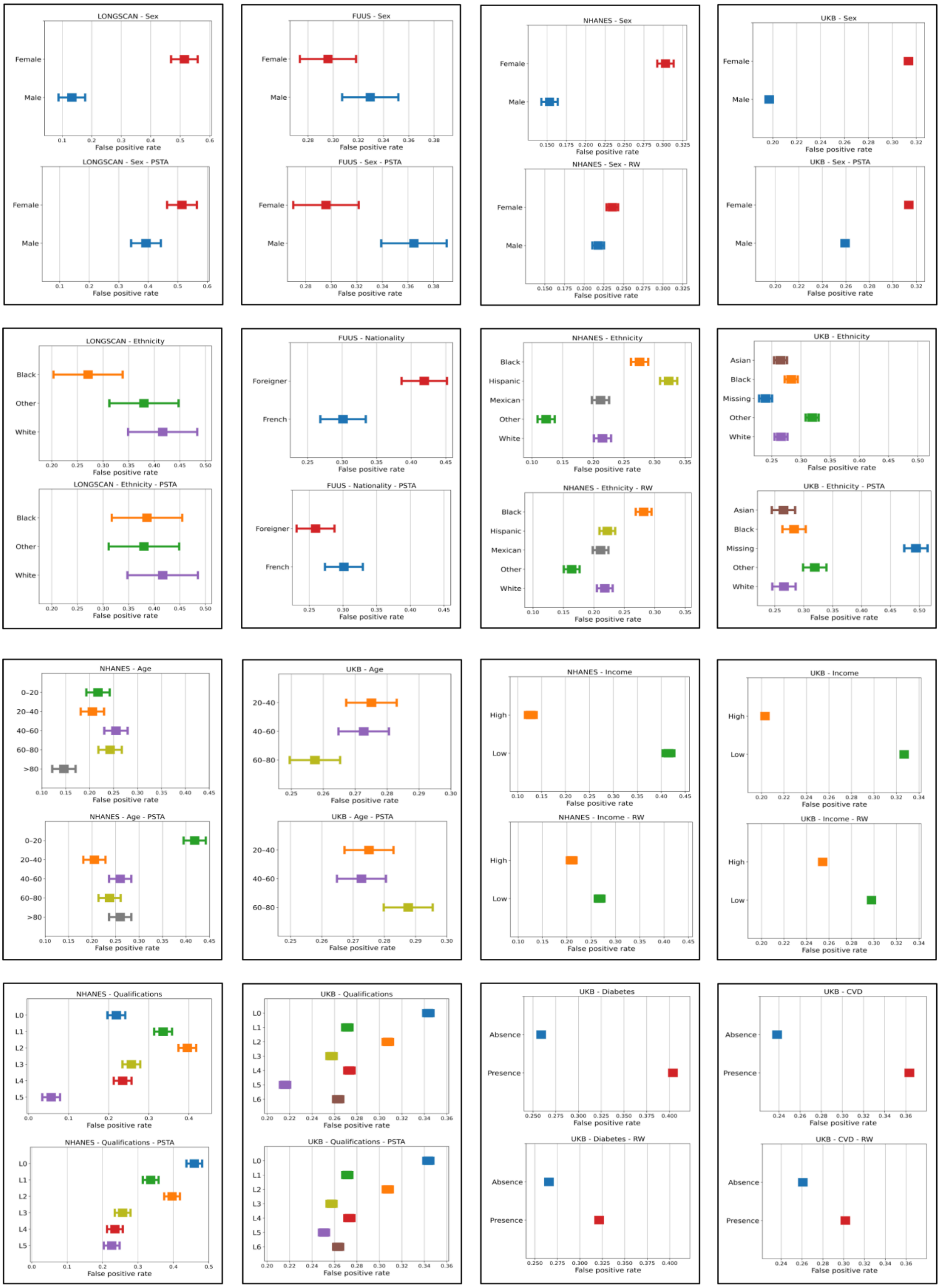

Supplementary Figure 6. Comparative analysis of group-specific FPRs for XGB classifiers: baseline and bias mitigation outcomes. Points represent mean FRP and error bars indicate 95% confidence intervals over k-fold cross-validation. Each plot represents a Dataset-Protected attribute pair, with paired rows displaying base classifiers and debiased classifiers, reporting the best results among the tested bias mitigation algorithms.

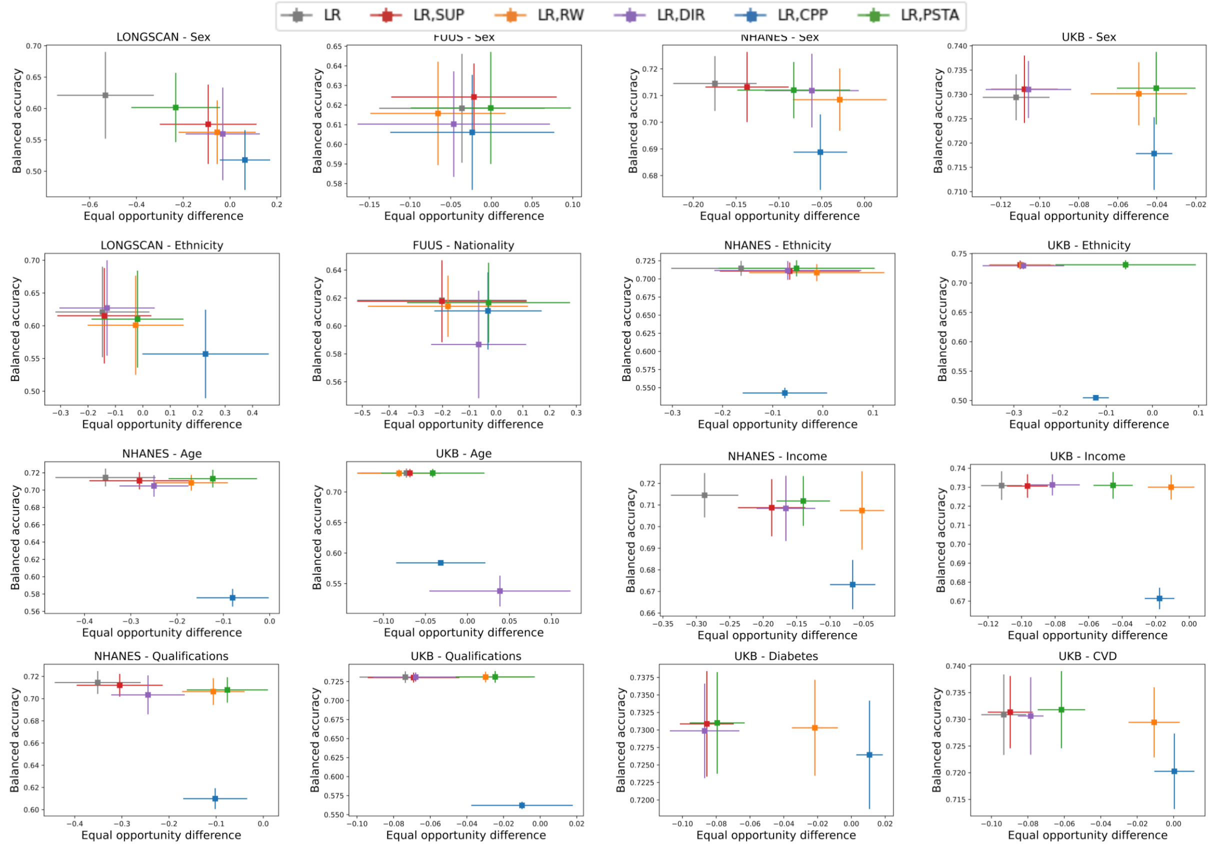

Supplementary Figure 7. Fairness-accuracy performance in terms of EOD vs. BAcc of the base model and the new classifiers after applying five bias mitigation algorithms to XGB classifiers. Points represent mean BAcc-TRP and error bars indicate the standard deviation over k-fold cross validation. Each plot shows the results per subgroup for a Dataset-Protected attribute pair.

Analysis of bias mitigation impact on untreated protected attributes

We consider each combination of *(dataset, ML model, treated protected attribute, and untreated protected attribute)* as a case and calculate the proportions of cases where a debiasing method, when targeting a specific protected attribute, inadvertently leads to a fairness decrease or increase for other unprotected attributes (measured by changes in EOD or AOD metrics). Our analysis reveals that for EOD and AOD, 45.5% and 42.7% of the total cases exhibit reduced fairness. Notably, the reduction is statistically significant in only 4.5% and 3.8% of cases for EOD and AOD, respectively, determined by the Mann-Whitney U-test (p-value < 0.05). For a comprehensive breakdown and further details of these findings, see Supplementary Table 12. These averaged preliminary results suggest that the trend toward worsening fairness on untreated attributes is not uniform, although this undesirable behavior happens frequently and deserves attention. Regarding the debiasing methods, in terms of both metrics, simple methods such as SUP seem more inclined to worsening, and postprocessing methods appear to be more robust against it.

Supplementary Table 12. Summary of preliminary multi-attribute fairness analysis per debiasing method and fairness metric. Each cell shows the percentage of cases, across four datasets and all considered protected attributes, exhibiting a worsening or an improvement in fairness on untreated attributes. In parentheses, it shows the proportion of statistically significant cases, as determined by the Mann-Whitney U-test (p-value < 0.05).

Method	EOD		AOD	
	% Worse (% Sig. Worse)	% Improved (% Sig. Improved)	% Worse (% Sig. Worse)	% Improved (% Sig. Improved)
SUP	56.8 (4.5)	43.2 (0.0)	49.2 (3.8)	50.8 (5.3)
RW	53.8 (3.8)	46.2 (3.0)	47.0 (5.3)	53.0 (13.6)
DIR	45.5 (3.8)	54.5 (3.9)	44.7 (0.8)	55.3 (6.1)
CPP	38.8 (6.8)	68.2 (38.6)	19.7 (6.1)	80.3 (49.2)
PSTA	39.4 (3.8)	60.6 (4.5)	53.0 (3.0)	47.0 (8.3)
Average across five methods	46.8 (4.5)	54.5 (10.0)	42.7 (3.8)	57.3 (16.5)
All cases	45.5 (4.5)	54.5 (10.0)	42.7 (3.8)	57.3 (16.5)

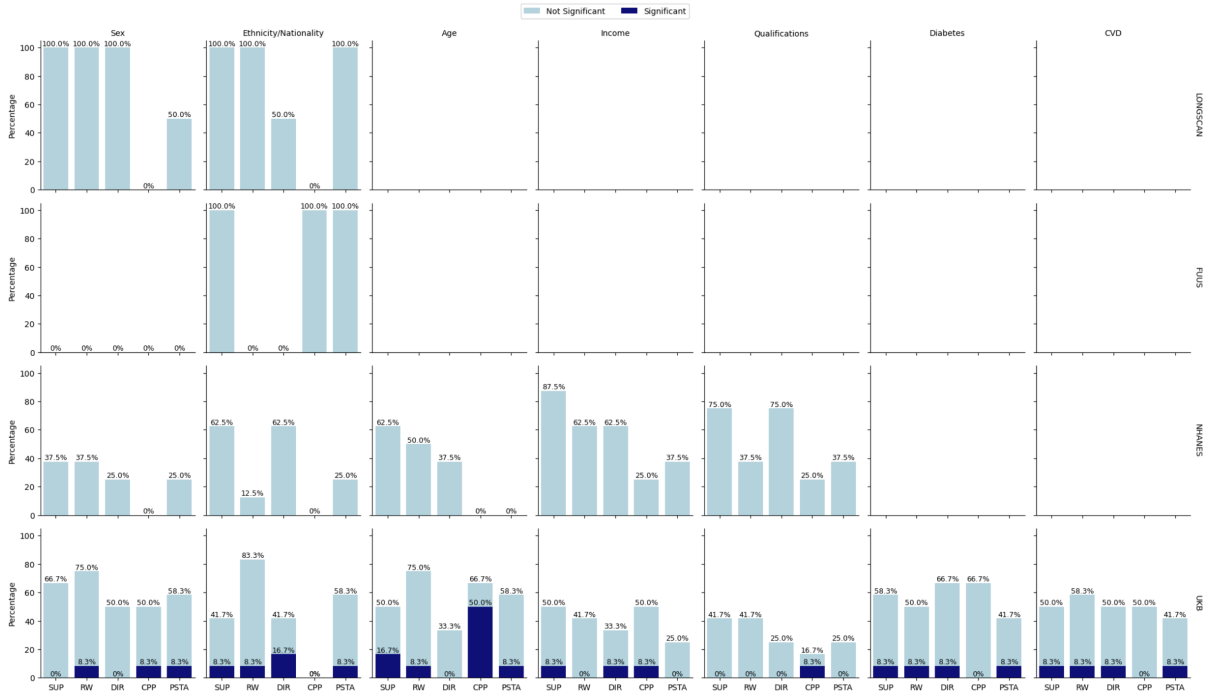

Supplementary Figure 8: Summary of preliminary multi-attribute fairness analysis per dataset (rows) and targeted protected attribute (columns). Each plot shows the proportion of cases where, with each debiasing method, targeting the corresponding protected attribute inadvertently reduces EOD fairness metric applied to two ML models (LR and XGB) for other (untreated) protected attributes. Dark-blue columns indicate the proportion of cases where the reduction is statistically significant, determined by the Mann-Whitney U-test (p-value < 0.05), whereas light-blue counts are non-significant. Empty plots indicate the absence of the corresponding dataset-attribute combination.

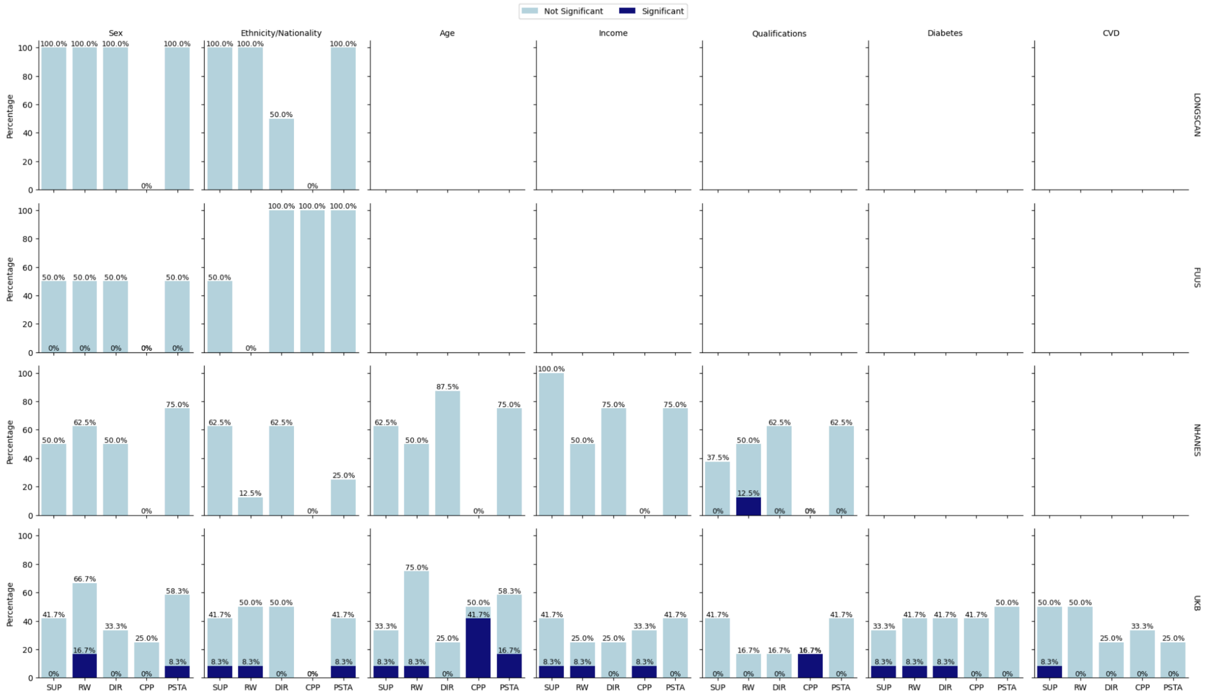

Supplementary Figure 9. Summary of preliminary multi-attribute fairness analysis per dataset (rows) and targeted protected attribute (columns). Each plot shows the proportion of cases where, with each debiasing method, targeting the corresponding protected attribute inadvertently reduces AOD fairness metric applied to two ML models (LR and XGB) for other (untreated) protected attributes. Dark-blue columns indicate the proportion of cases where the reduction is statistically significant, determined by the Mann-Whitney U-test (p-value < 0.05), whereas light-blue counts are non-significant. Empty plots indicate the absence of the corresponding dataset-attribute combination.

Supplementary Figures 8 and 9 depict our preliminary multi-attribute fairness analysis, broken down by dataset and targeted protected attribute in terms of EOD and AOD metrics, respectively. Notably, while only a small fraction of cases demonstrated a statistically significant reduction in fairness (p-value < 0.05), most of them occurred within the UKB dataset. Following the experimental design in [1], we also analyzed whether a possible correlation between targeted and untreated attributes is relevant to the impact on untreated attributes' fairness. Supplementary Figure 10 illustrates the pairwise correlation between protected attributes in the four cohorts used in this study on the mental health domain. In the UKB dataset, there is a higher prevalence of statistically significant negative Spearman correlation coefficients (p-value < 0.05) between pairs of protected attributes, indicating stronger inverse relationships in comparison to the other three datasets. This distribution aligns with the results presented in Supplementary Figures 8 and 9, illustrating that as fairness is enhanced for one attribute, it may inversely affect another if the attributes are negatively correlated.

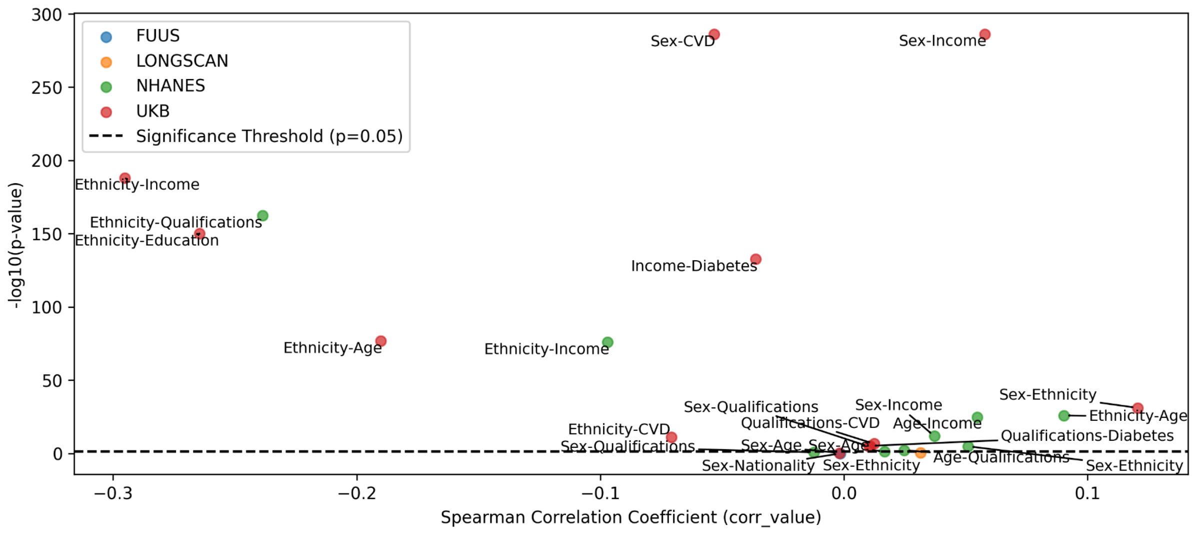

Supplementary Figure 10. Correlation of protected attributes in four study populations. The horizontal dashed line sets the significance threshold (p-value = 0.05).

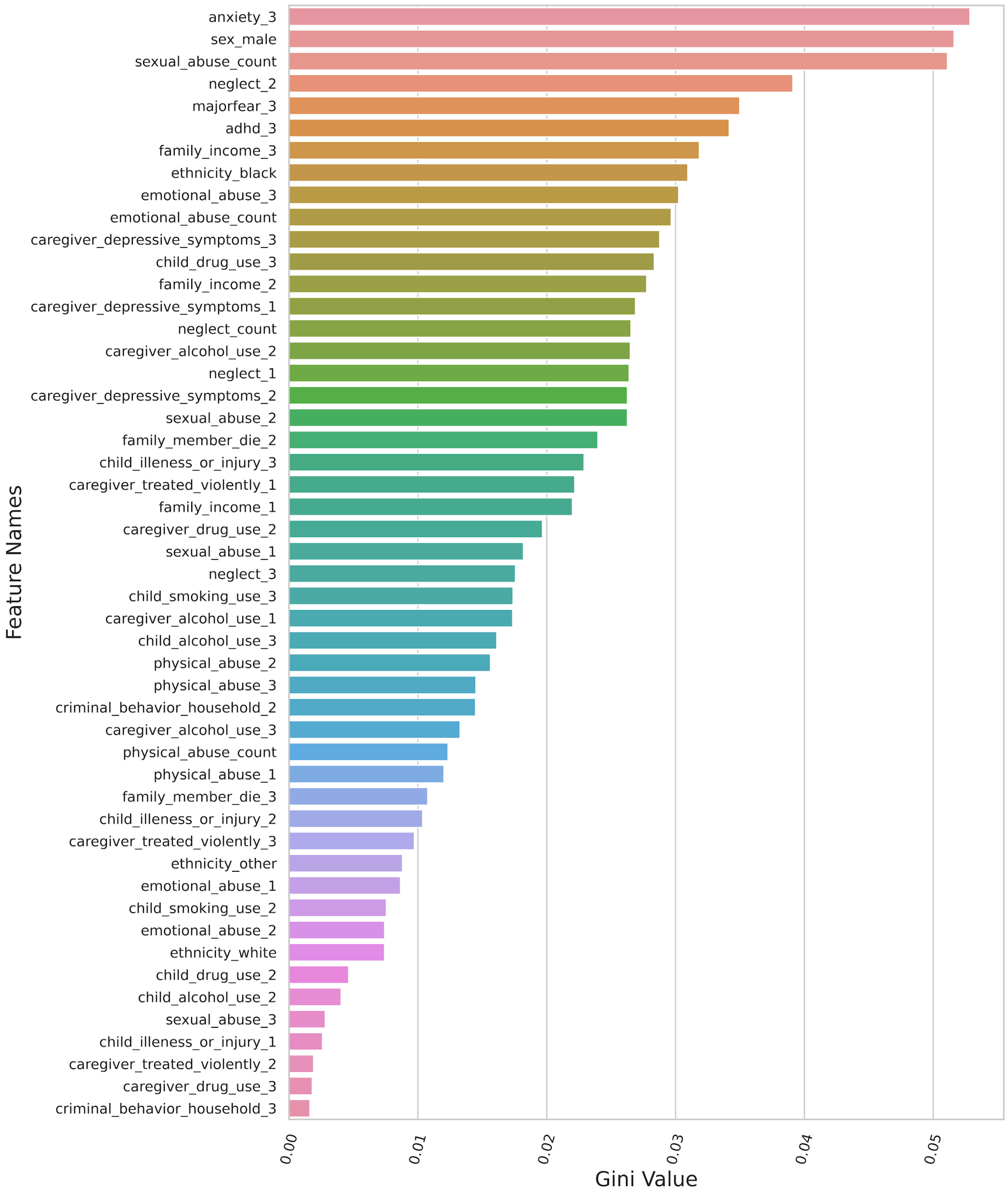

Supplementary Figure 11. LONGSCAN - Features with greatest Gini index values in the XGB model

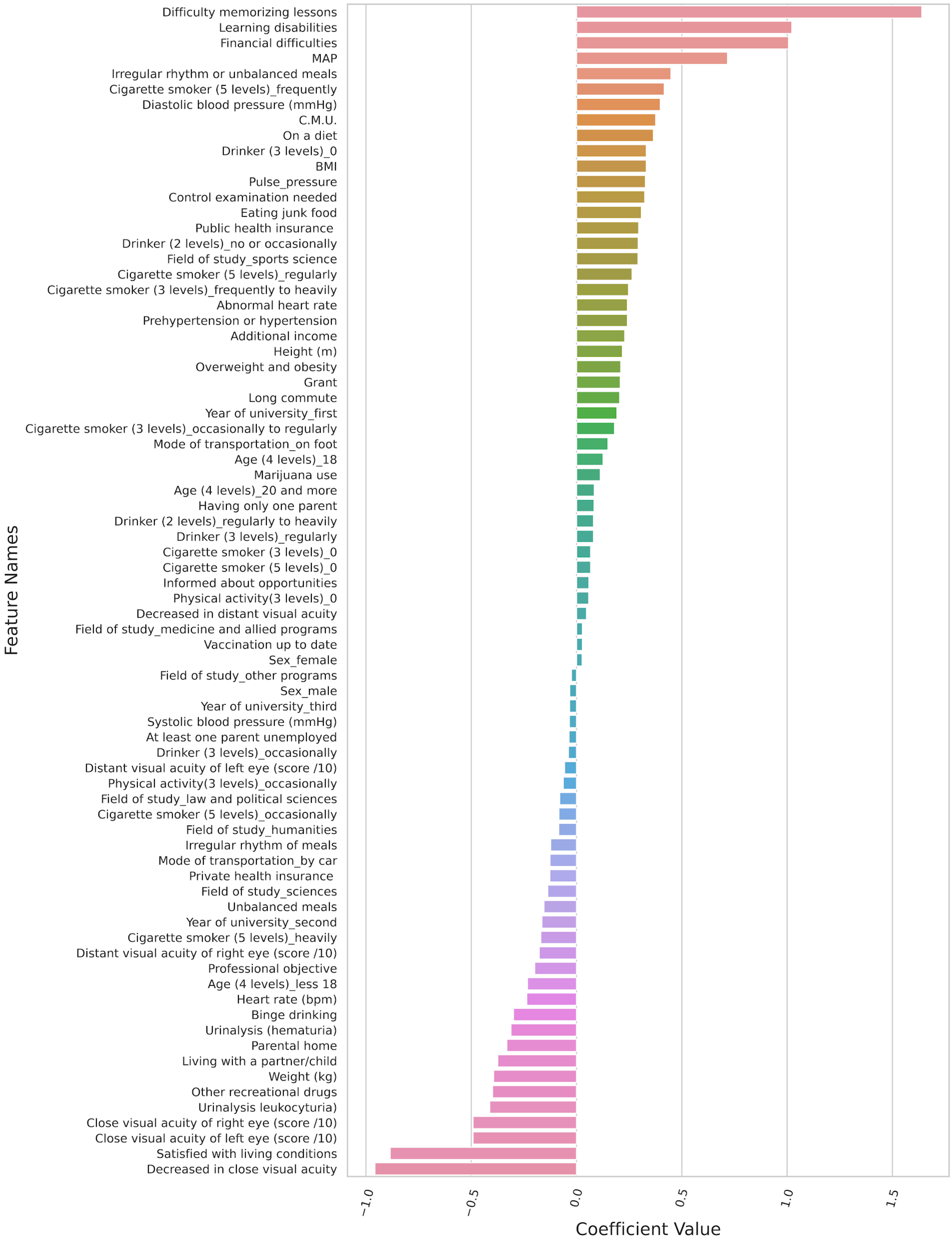

Supplementary Figure 12. FUUS - Features with greatest coefficient absolute values in the LR model. Note that a positive coefficient indicates that the event is more likely to occur, and a negative coefficient indicates that the event becomes less likely.

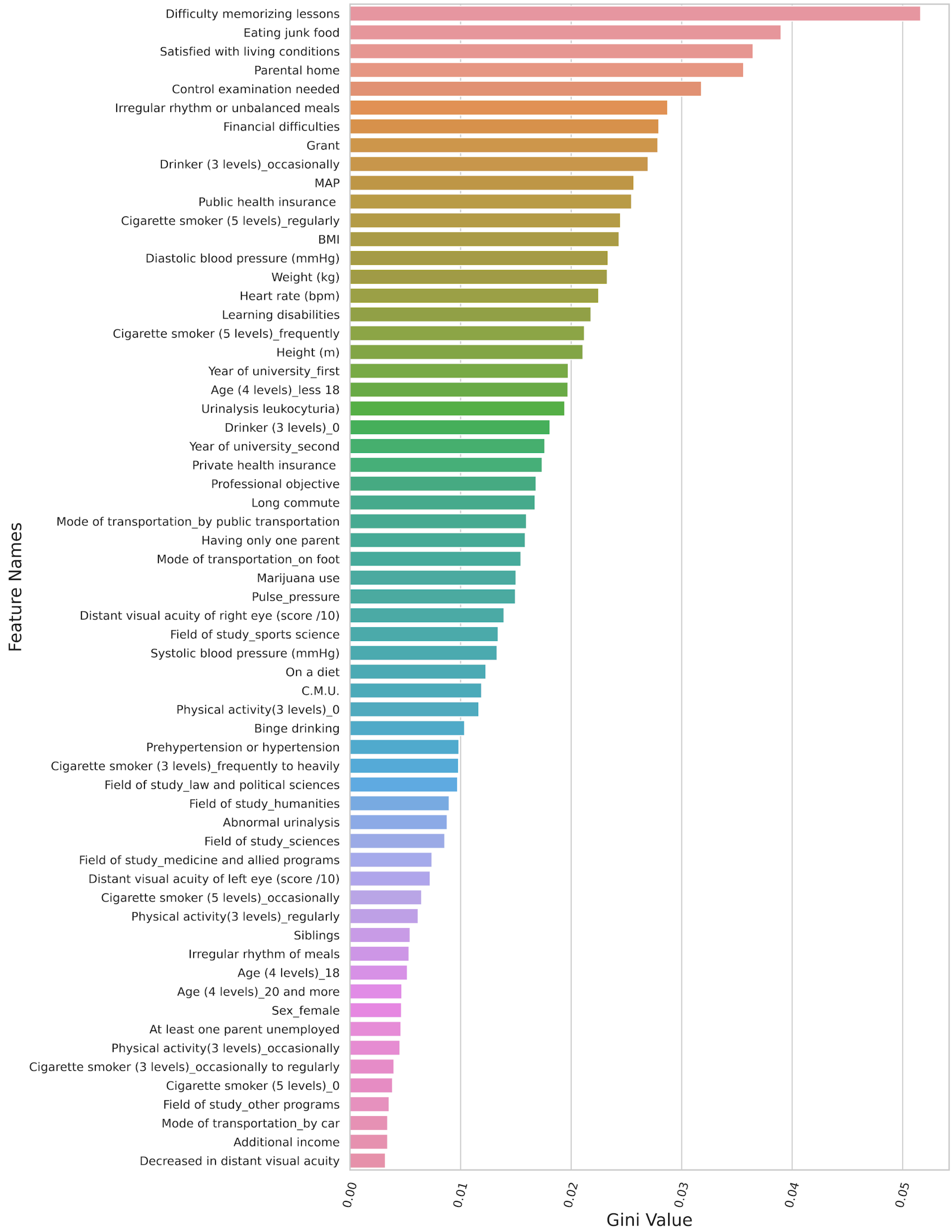

Supplementary Figure 13. FUUS - Features with greatest Gini index values in the XGB model

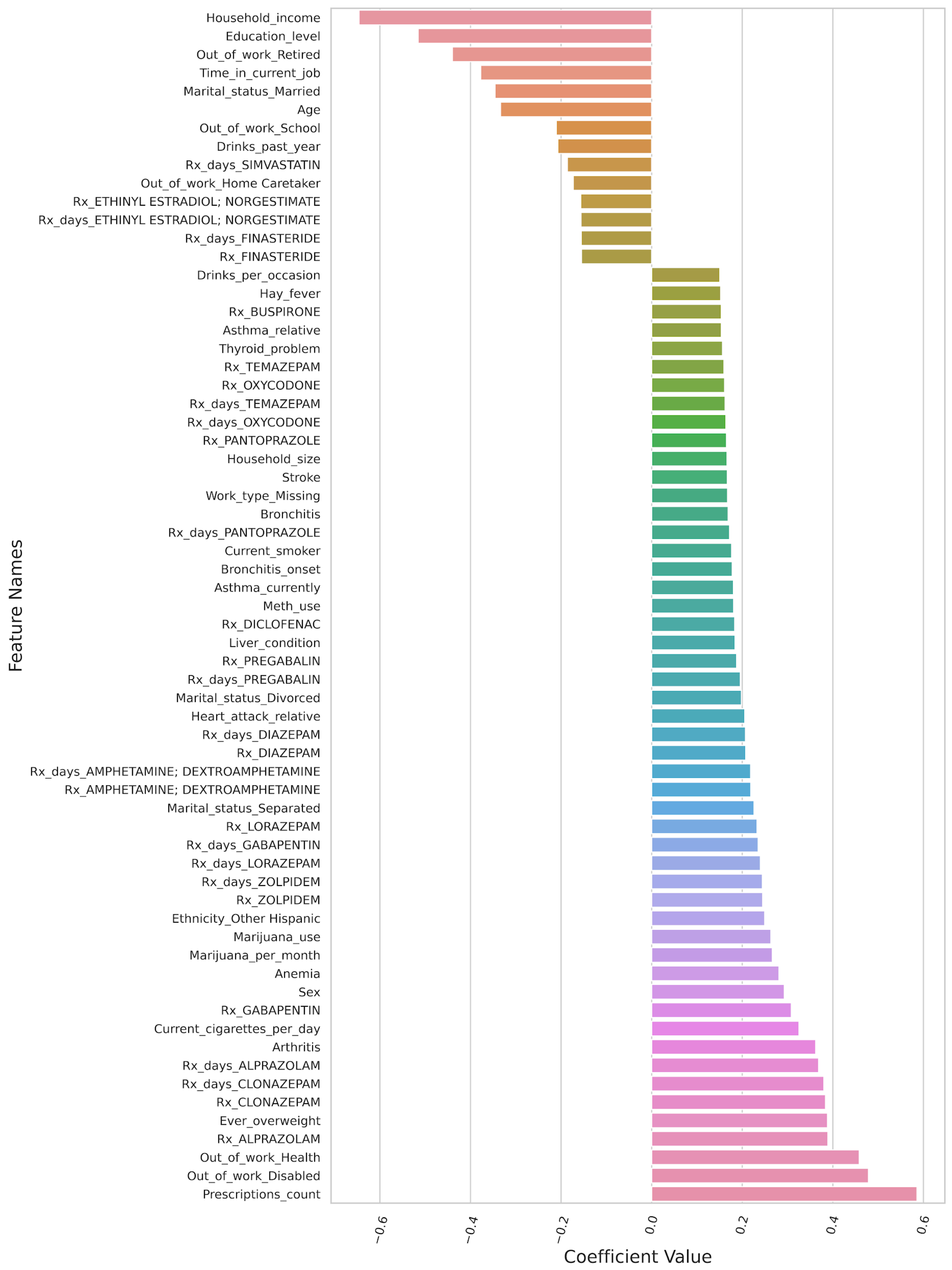

Supplementary Figure 14. NHANES - Features with greatest coefficient absolute values in the LR model. Note that a positive coefficient indicates that the event is more likely to occur, and a negative coefficient indicates that the event becomes less likely.

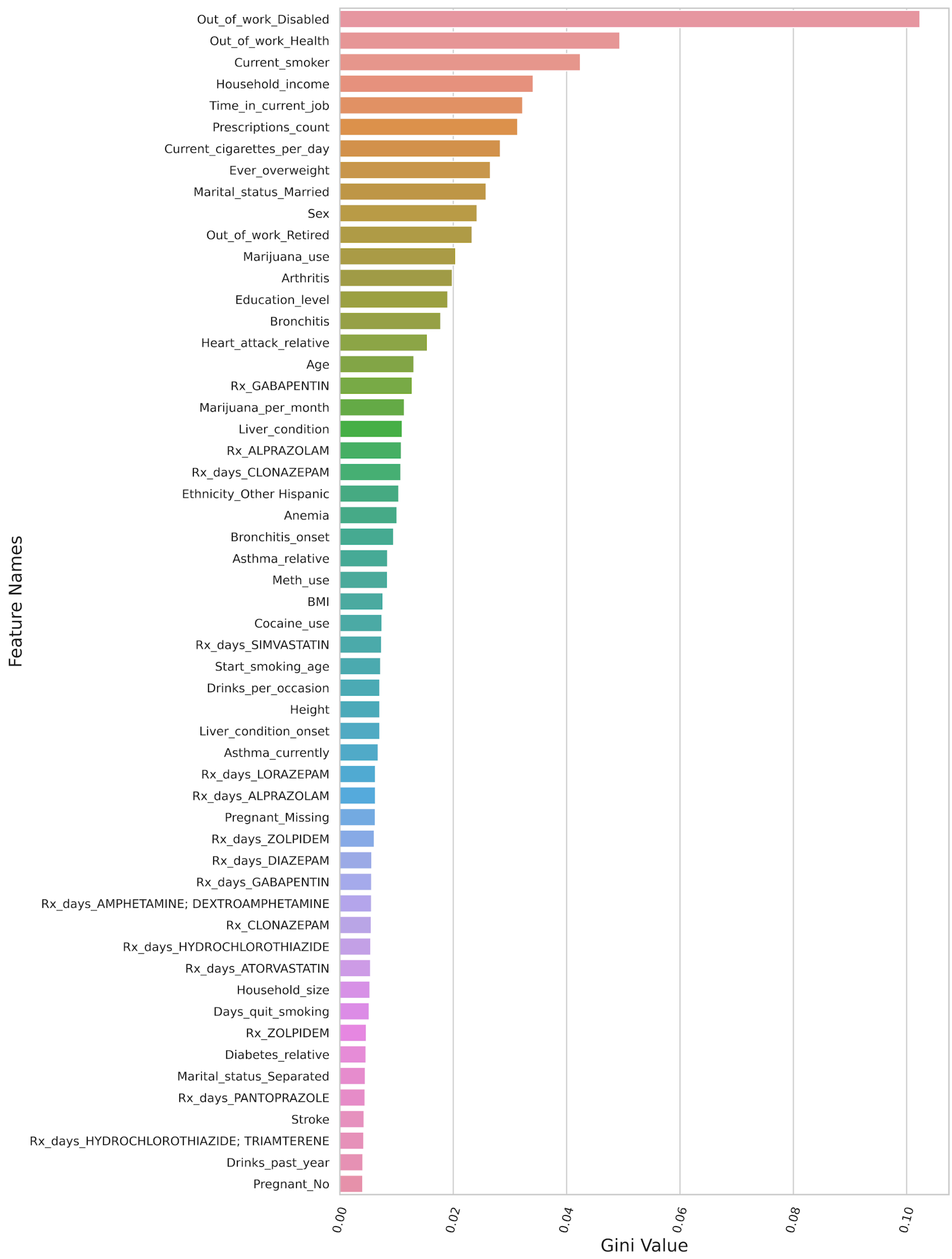

Supplementary Figure 15. NHANES - Features with greatest Gini index values in the XGB model

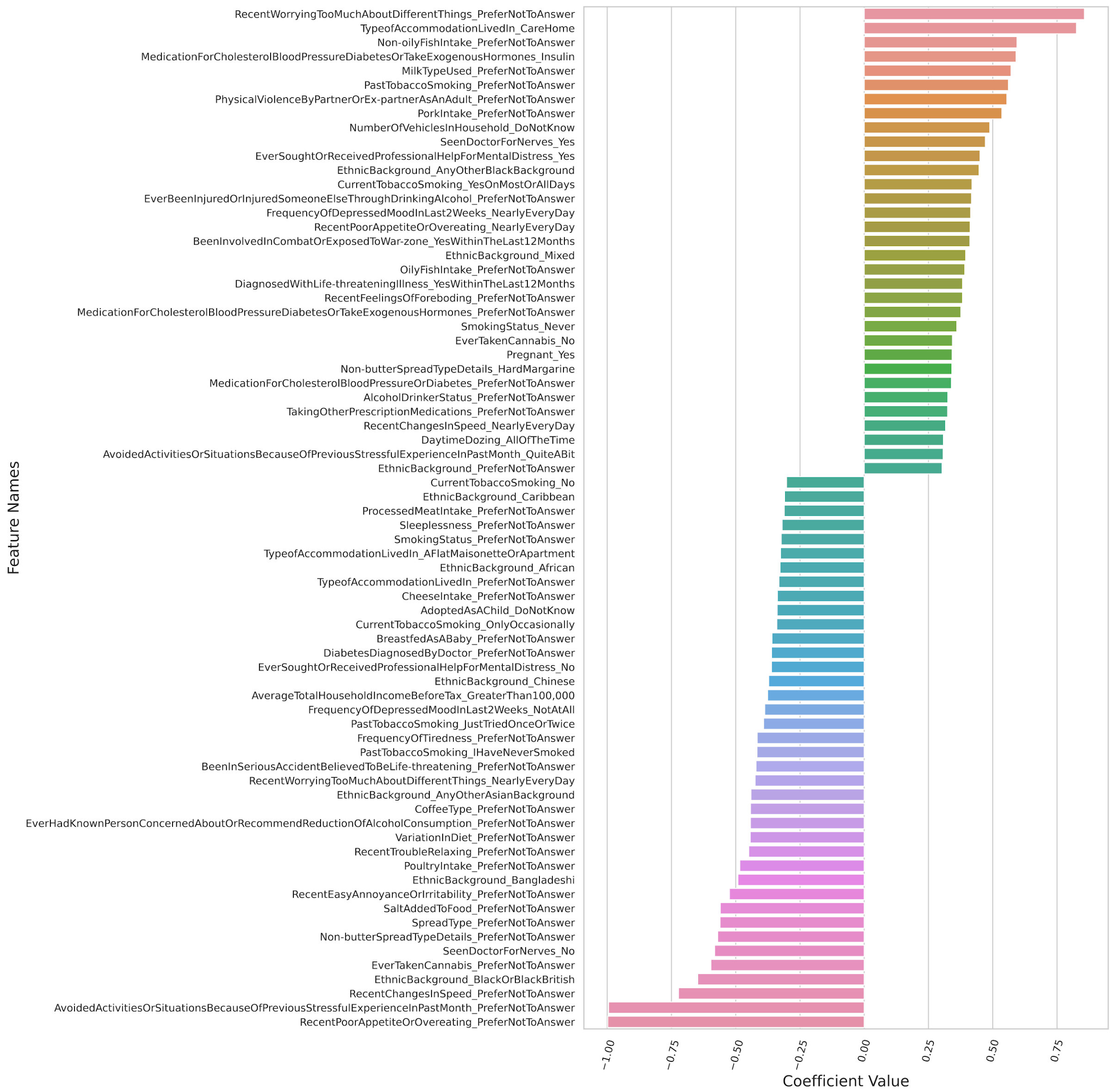

Supplementary Figure 16. UKB - Features with greatest coefficient absolute values in the LR model. Note that a positive coefficient indicates that the event is more likely to occur, and a negative coefficient indicates that the event becomes less likely.

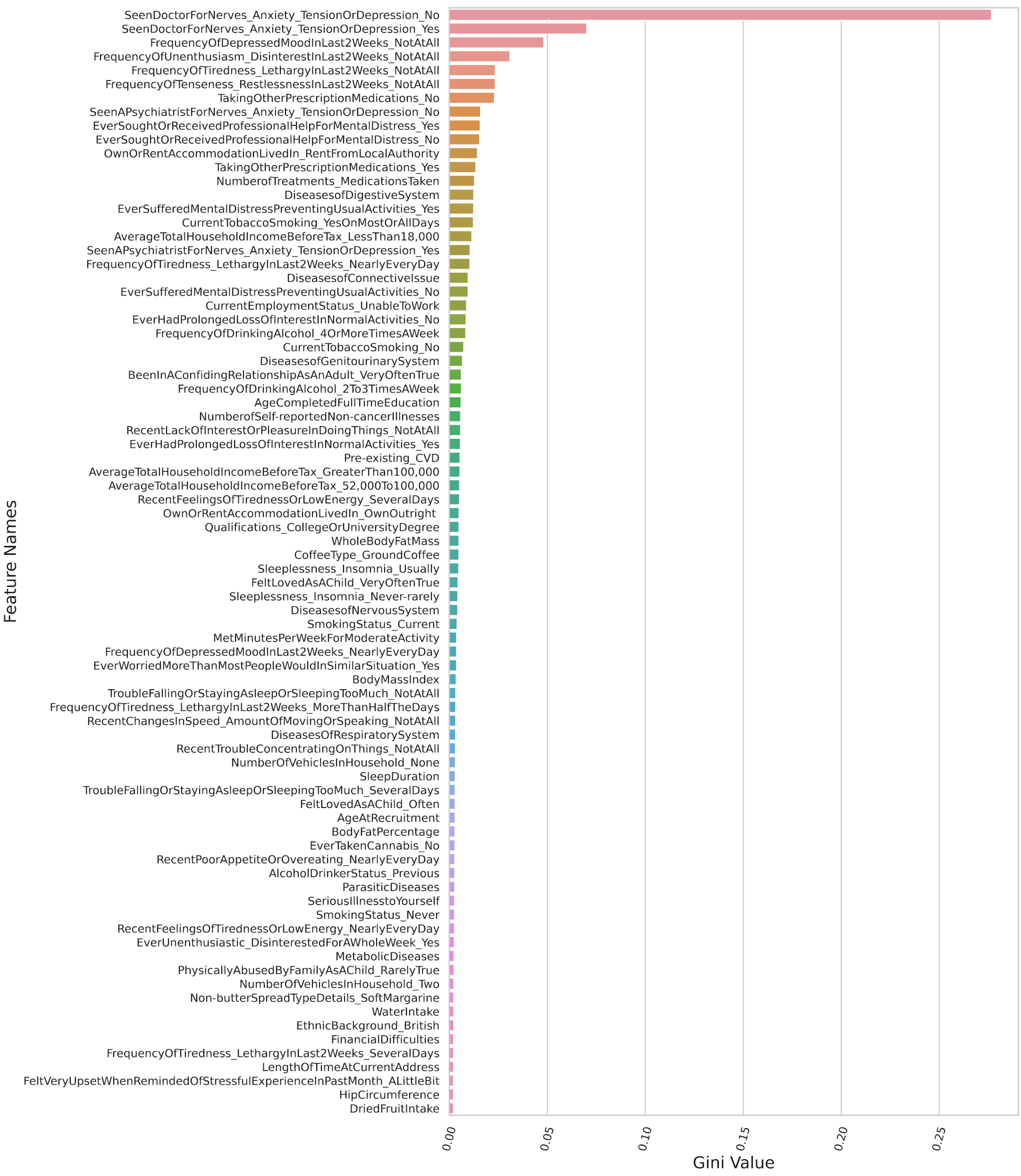

Supplementary Figure 17. UKB - Features with greatest Gini index values in the XGB model
